# Measurements of differential and double-differential Drell–Yan cross sections in proton–proton collisions at $$\sqrt{s} = 8$$$$\,\text {TeV}$$

**DOI:** 10.1140/epjc/s10052-015-3364-2

**Published:** 2015-04-09

**Authors:** V. Khachatryan, A. M. Sirunyan, A. Tumasyan, W. Adam, T. Bergauer, M. Dragicevic, J. Erö, M. Friedl, R. Frühwirth, V. M. Ghete, C. Hartl, N. Hörmann, J. Hrubec, M. Jeitler, W. Kiesenhofer, V. Knünz, M. Krammer, I. Krätschmer, D. Liko, I. Mikulec, D. Rabady, B. Rahbaran, H. Rohringer, R. Schöfbeck, J. Strauss, W. Treberer-Treberspurg, W. Waltenberger, C.-E. Wulz, V. Mossolov, N. Shumeiko, J. Suarez Gonzalez, S. Alderweireldt, S. Bansal, T. Cornelis, E. A. De Wolf, X. Janssen, A. Knutsson, J. Lauwers, S. Luyckx, S. Ochesanu, R. Rougny, M. Van De Klundert, H. Van Haevermaet, P. Van Mechelen, N. Van Remortel, A. Van Spilbeeck, F. Blekman, S. Blyweert, J. D’Hondt, N. Daci, N. Heracleous, J. Keaveney, S. Lowette, M. Maes, A. Olbrechts, Q. Python, D. Strom, S. Tavernier, W. Van Doninck, P. Van Mulders, G. P. Van Onsem, I. Villella, C. Caillol, B. Clerbaux, G. De Lentdecker, D. Dobur, L. Favart, A. P. R. Gay, A. Grebenyuk, A. Léonard, A. Mohammadi, L. Perniè, A. Randle-conde, T. Reis, T. Seva, L. Thomas, C. Vander Velde, P. Vanlaer, J. Wang, F. Zenoni, V. Adler, K. Beernaert, L. Benucci, A. Cimmino, S. Costantini, S. Crucy, S. Dildick, A. Fagot, G. Garcia, J. Mccartin, A. A. Ocampo Rios, D. Poyraz, D. Ryckbosch, S. Salva Diblen, M. Sigamani, N. Strobbe, F. Thyssen, M. Tytgat, E. Yazgan, N. Zaganidis, S. Basegmez, C. Beluffi, G. Bruno, R. Castello, A. Caudron, L. Ceard, G. G. Da Silveira, C. Delaere, T. du Pree, D. Favart, L. Forthomme, A. Giammanco, J. Hollar, A. Jafari, P. Jez, M. Komm, V. Lemaitre, C. Nuttens, L. Perrini, A. Pin, K. Piotrzkowski, A. Popov, L. Quertenmont, M. Selvaggi, M. Vidal Marono, J. M. Vizan Garcia, N. Beliy, T. Caebergs, E. Daubie, G. H. Hammad, W. L. Aldá Júnior, G. A. Alves, L. Brito, M. Correa Martins Junior, T. Dos Reis Martins, J. Molina, C. Mora Herrera, M. E. Pol, P. Rebello Teles, W. Carvalho, J. Chinellato, A. Custódio, E. M. Da Costa, D. De Jesus Damiao, C. De Oliveira Martins, S. Fonseca De Souza, H. Malbouisson, D. Matos Figueiredo, L. Mundim, H. Nogima, W. L. Prado Da Silva, J. Santaolalla, A. Santoro, A. Sznajder, E. J. Tonelli Manganote, A. Vilela Pereira, C. A. Bernardes, S. Dogra, T. R. Fernandez Perez Tomei, E. M. Gregores, P. G. Mercadante, S. F. Novaes, Sandra S. Padula, A. Aleksandrov, V. Genchev, R. Hadjiiska, P. Iaydjiev, A. Marinov, S. Piperov, M. Rodozov, S. Stoykova, G. Sultanov, M. Vutova, A. Dimitrov, I. Glushkov, L. Litov, B. Pavlov, P. Petkov, J. G. Bian, G. M. Chen, H. S. Chen, M. Chen, T. Cheng, R. Du, C. H. Jiang, R. Plestina, F. Romeo, J. Tao, Z. Wang, C. Asawatangtrakuldee, Y. Ban, Q. Li, S. Liu, Y. Mao, S. J. Qian, D. Wang, Z. Xu, W. Zou, C. Avila, A. Cabrera, L. F. Chaparro Sierra, C. Florez, J. P. Gomez, B. Gomez Moreno, J. C. Sanabria, N. Godinovic, D. Lelas, D. Polic, I. Puljak, Z. Antunovic, M. Kovac, V. Brigljevic, K. Kadija, J. Luetic, D. Mekterovic, L. Sudic, A. Attikis, G. Mavromanolakis, J. Mousa, C. Nicolaou, F. Ptochos, P. A. Razis, M. Bodlak, M. Finger, M. Finger, Y. Assran, A. Ellithi Kamel, M. A. Mahmoud, A. Radi, M. Kadastik, M. Murumaa, M. Raidal, A. Tiko, P. Eerola, M. Voutilainen, J. Härkönen, V. Karimäki, R. Kinnunen, M. J. Kortelainen, T. Lampén, K. Lassila-Perini, S. Lehti, T. Lindén, P. Luukka, T. Mäenpää, T. Peltola, E. Tuominen, J. Tuominiemi, E. Tuovinen, L. Wendland, J. Talvitie, T. Tuuva, M. Besancon, F. Couderc, M. Dejardin, D. Denegri, B. Fabbro, J. L. Faure, C. Favaro, F. Ferri, S. Ganjour, A. Givernaud, P. Gras, G. Hamel de Monchenault, P. Jarry, E. Locci, J. Malcles, J. Rander, A. Rosowsky, M. Titov, S. Baffioni, F. Beaudette, P. Busson, E. Chapon, C. Charlot, T. Dahms, M. Dalchenko, L. Dobrzynski, N. Filipovic, A. Florent, R. Granier de Cassagnac, L. Mastrolorenzo, P. Miné, I. N. Naranjo, M. Nguyen, C. Ochando, G. Ortona, P. Paganini, S. Regnard, R. Salerno, J. B. Sauvan, Y. Sirois, C. Veelken, Y. Yilmaz, A. Zabi, J.-L. Agram, J. Andrea, A. Aubin, D. Bloch, J.-M. Brom, E. C. Chabert, C. Collard, E. Conte, J.-C. Fontaine, D. Gelé, U. Goerlach, C. Goetzmann, A.-C. Le Bihan, K. Skovpen, P. Van Hove, S. Gadrat, S. Beauceron, N. Beaupere, C. Bernet, G. Boudoul, E. Bouvier, S. Brochet, C. A. Carrillo Montoya, J. Chasserat, R. Chierici, D. Contardo, P. Depasse, H. El Mamouni, J. Fan, J. Fay, S. Gascon, M. Gouzevitch, B. Ille, T. Kurca, M. Lethuillier, L. Mirabito, S. Perries, J. D. Ruiz Alvarez, D. Sabes, L. Sgandurra, V. Sordini, M. Vander Donckt, P. Verdier, S. Viret, H. Xiao, Z. Tsamalaidze, C. Autermann, S. Beranek, M. Bontenackels, M. Edelhoff, L. Feld, A. Heister, K. Klein, M. Lipinski, A. Ostapchuk, M. Preuten, F. Raupach, J. Sammet, S. Schael, J. F. Schulte, H. Weber, B. Wittmer, V. Zhukov, M. Ata, M. Brodski, E. Dietz-Laursonn, D. Duchardt, M. Erdmann, R. Fischer, A. Güth, T. Hebbeker, C. Heidemann, K. Hoepfner, D. Klingebiel, S. Knutzen, P. Kreuzer, M. Merschmeyer, A. Meyer, G. Mittag, P. Millet, M. Olschewski, K. Padeken, P. Papacz, H. Reithler, S. A. Schmitz, L. Sonnenschein, D. Teyssier, S. Thüer, M. Weber, V. Cherepanov, Y. Erdogan, G. Flügge, H. Geenen, M. Geisler, W. Haj Ahmad, F. Hoehle, B. Kargoll, T. Kress, Y. Kuessel, A. Künsken, J. Lingemann, A. Nowack, I. M. Nugent, O. Pooth, A. Stahl, M. Aldaya Martin, I. Asin, N. Bartosik, J. Behr, U. Behrens, A. J. Bell, A. Bethani, K. Borras, A. Burgmeier, A. Cakir, L. Calligaris, A. Campbell, S. Choudhury, F. Costanza, C. Diez Pardos, G. Dolinska, S. Dooling, T. Dorland, G. Eckerlin, D. Eckstein, T. Eichhorn, G. Flucke, J. Garay Garcia, A. Geiser, P. Gunnellini, J. Hauk, M. Hempel, H. Jung, A. Kalogeropoulos, M. Kasemann, P. Katsas, J. Kieseler, C. Kleinwort, I. Korol, D. Krücker, W. Lange, J. Leonard, K. Lipka, A. Lobanov, W. Lohmann, B. Lutz, R. Mankel, I. Marfin, I.-A. Melzer-Pellmann, A. B. Meyer, J. Mnich, A. Mussgiller, S. Naumann-Emme, A. Nayak, E. Ntomari, H. Perrey, D. Pitzl, R. Placakyte, A. Raspereza, P. M. Ribeiro Cipriano, B. Roland, E. Ron, M. Ö. Sahin, J. Salfeld-Nebgen, P. Saxena, T. Schoerner-Sadenius, M. Schröder, C. Seitz, S. Spannagel, A. D. R. Vargas Trevino, R. Walsh, C. Wissing, V. Blobel, M. Centis Vignali, A. R. Draeger, J. Erfle, E. Garutti, K. Goebel, M. Görner, J. Haller, M. Hoffmann, R. S. Höing, A. Junkes, H. Kirschenmann, R. Klanner, R. Kogler, J. Lange, T. Lapsien, T. Lenz, I. Marchesini, J. Ott, T. Peiffer, A. Perieanu, N. Pietsch, J. Poehlsen, T. Poehlsen, D. Rathjens, C. Sander, H. Schettler, P. Schleper, E. Schlieckau, A. Schmidt, M. Seidel, V. Sola, H. Stadie, G. Steinbrück, D. Troendle, E. Usai, L. Vanelderen, A. Vanhoefer, C. Barth, C. Baus, J. Berger, C. Böser, E. Butz, T. Chwalek, W. De Boer, A. Descroix, A. Dierlamm, M. Feindt, F. Frensch, M. Giffels, A. Gilbert, F. Hartmann, T. Hauth, U. Husemann, I. Katkov, A. Kornmayer, P. Lobelle Pardo, M. U. Mozer, T. Müller, Th. Müller, A. Nürnberg, G. Quast, K. Rabbertz, S. Röcker, H. J. Simonis, F. M. Stober, R. Ulrich, J. Wagner-Kuhr, S. Wayand, T. Weiler, R. Wolf, G. Anagnostou, G. Daskalakis, T. Geralis, V. A. Giakoumopoulou, A. Kyriakis, D. Loukas, A. Markou, C. Markou, A. Psallidas, I. Topsis-Giotis, A. Agapitos, S. Kesisoglou, A. Panagiotou, N. Saoulidou, E. Stiliaris, X. Aslanoglou, I. Evangelou, G. Flouris, C. Foudas, P. Kokkas, N. Manthos, I. Papadopoulos, J. Strologas, E. Paradas, G. Bencze, C. Hajdu, P. Hidas, D. Horvath, F. Sikler, V. Veszpremi, G. Vesztergombi, A. J. Zsigmond, N. Beni, S. Czellar, J. Karancsi, J. Molnar, J. Palinkas, Z. Szillasi, A. Makovec, P. Raics, Z. L. Trocsanyi, B. Ujvari, S. K. Swain, S. B. Beri, V. Bhatnagar, R. Gupta, U. Bhawandeep, A. K. Kalsi, M. Kaur, R. Kumar, M. Mittal, N. Nishu, J. B. Singh, Ashok Kumar, Arun Kumar, S. Ahuja, A. Bhardwaj, B. C. Choudhary, A. Kumar, S. Malhotra, M. Naimuddin, K. Ranjan, V. Sharma, S. Banerjee, S. Bhattacharya, K. Chatterjee, S. Dutta, B. Gomber, Sa. Jain, Sh. Jain, R. Khurana, A. Modak, S. Mukherjee, D. Roy, S. Sarkar, M. Sharan, A. Abdulsalam, D. Dutta, V. Kumar, A. K. Mohanty, L. M. Pant, P. Shukla, A. Topkar, T. Aziz, S. Banerjee, S. Bhowmik, R. M. Chatterjee, R. K. Dewanjee, S. Dugad, S. Ganguly, S. Ghosh, M. Guchait, A. Gurtu, G. Kole, S. Kumar, M. Maity, G. Majumder, K. Mazumdar, G. B. Mohanty, B. Parida, K. Sudhakar, N. Wickramage, H. Bakhshiansohi, H. Behnamian, S. M. Etesami, A. Fahim, R. Goldouzian, M. Khakzad, M. Mohammadi Najafabadi, M. Naseri, S. Paktinat Mehdiabadi, F. Rezaei Hosseinabadi, B. Safarzadeh, M. Zeinali, M. Felcini, M. Grunewald, M. Abbrescia, C. Calabria, S. S. Chhibra, A. Colaleo, D. Creanza, N. De Filippis, M. De Palma, L. Fiore, G. Iaselli, G. Maggi, M. Maggi, S. My, S. Nuzzo, A. Pompili, G. Pugliese, R. Radogna, G. Selvaggi, A. Sharma, L. Silvestris, R. Venditti, P. Verwilligen, G. Abbiendi, A. C. Benvenuti, D. Bonacorsi, S. Braibant-Giacomelli, L. Brigliadori, R. Campanini, P. Capiluppi, A. Castro, F. R. Cavallo, G. Codispoti, M. Cuffiani, G. M. Dallavalle, F. Fabbri, A. Fanfani, D. Fasanella, P. Giacomelli, C. Grandi, L. Guiducci, S. Marcellini, G. Masetti, A. Montanari, F. L. Navarria, A. Perrotta, F. Primavera, A. M. Rossi, T. Rovelli, G. P. Siroli, N. Tosi, R. Travaglini, S. Albergo, G. Cappello, M. Chiorboli, S. Costa, F. Giordano, R. Potenza, A. Tricomi, C. Tuve, G. Barbagli, V. Ciulli, C. Civinini, R. D’Alessandro, E. Focardi, E. Gallo, S. Gonzi, V. Gori, P. Lenzi, M. Meschini, S. Paoletti, G. Sguazzoni, A. Tropiano, L. Benussi, S. Bianco, F. Fabbri, D. Piccolo, R. Ferretti, F. Ferro, M. Lo Vetere, E. Robutti, S. Tosi, M. E. Dinardo, S. Fiorendi, S. Gennai, R. Gerosa, A. Ghezzi, P. Govoni, M. T. Lucchini, S. Malvezzi, R. A. Manzoni, A. Martelli, B. Marzocchi, D. Menasce, L. Moroni, M. Paganoni, D. Pedrini, S. Ragazzi, N. Redaelli, T. Tabarelli de Fatis, S. Buontempo, N. Cavallo, S. Di Guida, F. Fabozzi, A. O. M. Iorio, L. Lista, S. Meola, M. Merola, P. Paolucci, P. Azzi, N. Bacchetta, M. Bellato, M. Biasotto, A. Branca, M. Dall’Osso, T. Dorigo, S. Fantinel, F. Fanzago, M. Galanti, F. Gasparini, A. Gozzelino, K. Kanishchev, S. Lacaprara, M. Margoni, A. T. Meneguzzo, J. Pazzini, N. Pozzobon, P. Ronchese, F. Simonetto, E. Torassa, M. Tosi, S. Vanini, P. Zotto, A. Zucchetta, G. Zumerle, M. Gabusi, S. P. Ratti, V. Re, C. Riccardi, P. Salvini, P. Vitulo, M. Biasini, G. M. Bilei, D. Ciangottini, L. Fanò, P. Lariccia, G. Mantovani, M. Menichelli, A. Saha, A. Santocchia, A. Spiezia, K. Androsov, P. Azzurri, G. Bagliesi, J. Bernardini, T. Boccali, G. Broccolo, R. Castaldi, M. A. Ciocci, R. Dell’Orso, S. Donato, G. Fedi, F. Fiori, L. Foà, A. Giassi, M. T. Grippo, F. Ligabue, T. Lomtadze, L. Martini, A. Messineo, C. S. Moon, F. Palla, A. Rizzi, A. Savoy-Navarro, A. T. Serban, P. Spagnolo, P. Squillacioti, R. Tenchini, G. Tonelli, A. Venturi, P. G. Verdini, C. Vernieri, L. Barone, F. Cavallari, G. D’imperio, D. Del Re, M. Diemoz, C. Jorda, E. Longo, F. Margaroli, P. Meridiani, F. Micheli, G. Organtini, R. Paramatti, S. Rahatlou, C. Rovelli, F. Santanastasio, L. Soffi, P. Traczyk, N. Amapane, R. Arcidiacono, S. Argiro, M. Arneodo, R. Bellan, C. Biino, N. Cartiglia, S. Casasso, M. Costa, A. Degano, N. Demaria, L. Finco, C. Mariotti, S. Maselli, E. Migliore, V. Monaco, M. Musich, M. M. Obertino, L. Pacher, N. Pastrone, M. Pelliccioni, G. L. Pinna Angioni, A. Potenza, A. Romero, M. Ruspa, R. Sacchi, A. Solano, A. Staiano, U. Tamponi, S. Belforte, V. Candelise, M. Casarsa, F. Cossutti, G. Della Ricca, B. Gobbo, C. La Licata, M. Marone, A. Schizzi, T. Umer, A. Zanetti, S. Chang, A. Kropivnitskaya, S. K. Nam, D. H. Kim, G. N. Kim, M. S. Kim, M. S. Kim, D. J. Kong, S. Lee, Y. D. Oh, H. Park, A. Sakharov, D. C. Son, T. J. Kim, M. S. Ryu, J. Y. Kim, D. H. Moon, S. Song, S. Choi, D. Gyun, B. Hong, M. Jo, H. Kim, Y. Kim, B. Lee, K. S. Lee, S. K. Park, Y. Roh, H. D. Yoo, M. Choi, J. H. Kim, I. C. Park, G. Ryu, Y. Choi, Y. K. Choi, J. Goh, D. Kim, E. Kwon, J. Lee, I. Yu, A. Juodagalvis, J. R. Komaragiri, M. A. B. Md Ali, E. Casimiro Linares, H. Castilla-Valdez, E. De La Cruz-Burelo, I. Heredia-de La Cruz, A. Hernandez-Almada, R. Lopez-Fernandez, A. Sanchez-Hernandez, S. Carrillo Moreno, F. Vazquez Valencia, I. Pedraza, H. A. Salazar Ibarguen, A. Morelos Pineda, D. Krofcheck, P. H. Butler, S. Reucroft, A. Ahmad, M. Ahmad, Q. Hassan, H. R. Hoorani, W. A. Khan, T. Khurshid, M. Shoaib, H. Bialkowska, M. Bluj, B. Boimska, T. Frueboes, M. Górski, M. Kazana, K. Nawrocki, K. Romanowska-Rybinska, M. Szleper, P. Zalewski, G. Brona, K. Bunkowski, M. Cwiok, W. Dominik, K. Doroba, A. Kalinowski, M. Konecki, J. Krolikowski, M. Misiura, M. Olszewski, P. Bargassa, C. Beirão Da Cruz E Silva, P. Faccioli, P. G. Ferreira Parracho, M. Gallinaro, L. Lloret Iglesias, F. Nguyen, J. Rodrigues Antunes, J. Seixas, J. Varela, P. Vischia, S. Afanasiev, P. Bunin, M. Gavrilenko, I. Golutvin, I. Gorbunov, A. Kamenev, V. Karjavin, V. Konoplyanikov, A. Lanev, A. Malakhov, V. Matveev, P. Moisenz, V. Palichik, V. Perelygin, S. Shmatov, N. Skatchkov, V. Smirnov, A. Zarubin, V. Golovtsov, Y. Ivanov, V. Kim, E. Kuznetsova, P. Levchenko, V. Murzin, V. Oreshkin, I. Smirnov, V. Sulimov, L. Uvarov, S. Vavilov, A. Vorobyev, An. Vorobyev, Yu. Andreev, A. Dermenev, S. Gninenko, N. Golubev, M. Kirsanov, N. Krasnikov, A. Pashenkov, D. Tlisov, A. Toropin, V. Epshteyn, V. Gavrilov, N. Lychkovskaya, V. Popov, I. Pozdnyakov, G. Safronov, S. Semenov, A. Spiridonov, V. Stolin, E. Vlasov, A. Zhokin, V. Andreev, M. Azarkin, I. Dremin, M. Kirakosyan, A. Leonidov, G. Mesyats, S. V. Rusakov, A. Vinogradov, A. Belyaev, E. Boos, V. Bunichev, M. Dubinin, L. Dudko, A. Ershov, V. Klyukhin, O. Kodolova, I. Lokhtin, S. Obraztsov, M. Perfilov, V. Savrin, A. Snigirev, I. Azhgirey, I. Bayshev, S. Bitioukov, V. Kachanov, A. Kalinin, D. Konstantinov, V. Krychkine, V. Petrov, R. Ryutin, A. Sobol, L. Tourtchanovitch, S. Troshin, N. Tyurin, A. Uzunian, A. Volkov, P. Adzic, M. Ekmedzic, J. Milosevic, V. Rekovic, J. Alcaraz Maestre, C. Battilana, E. Calvo, M. Cerrada, M. Chamizo Llatas, N. Colino, B. De La Cruz, A. Delgado Peris, D. Domínguez Vázquez, A. Escalante Del Valle, C. Fernandez Bedoya, J. P. Fernández Ramos, J. Flix, M. C. Fouz, P. Garcia-Abia, O. Gonzalez Lopez, S. Goy Lopez, J. M. Hernandez, M. I. Josa, E. Navarro De Martino, A. Pérez-Calero Yzquierdo, J. Puerta Pelayo, A. Quintario Olmeda, I. Redondo, L. Romero, M. S. Soares, C. Albajar, J. F. de Trocóniz, M. Missiroli, D. Moran, H. Brun, J. Cuevas, J. Fernandez Menendez, S. Folgueras, I. Gonzalez Caballero, J. A. Brochero Cifuentes, I. J. Cabrillo, A. Calderon, J. Duarte Campderros, M. Fernandez, G. Gomez, A. Graziano, A. Lopez Virto, J. Marco, R. Marco, C. Martinez Rivero, F. Matorras, F. J. Munoz Sanchez, J. Piedra Gomez, T. Rodrigo, A. Y. Rodríguez-Marrero, A. Ruiz-Jimeno, L. Scodellaro, I. Vila, R. Vilar Cortabitarte, D. Abbaneo, E. Auffray, G. Auzinger, M. Bachtis, P. Baillon, A. H. Ball, D. Barney, A. Benaglia, J. Bendavid, L. Benhabib, J. F. Benitez, P. Bloch, A. Bocci, A. Bonato, O. Bondu, C. Botta, H. Breuker, T. Camporesi, G. Cerminara, S. Colafranceschi, M. D’Alfonso, D. d’Enterria, A. Dabrowski, A. David, F. De Guio, A. De Roeck, S. De Visscher, E. Di Marco, M. Dobson, M. Dordevic, B. Dorney, N. Dupont-Sagorin, A. Elliott-Peisert, G. Franzoni, W. Funk, D. Gigi, K. Gill, D. Giordano, M. Girone, F. Glege, R. Guida, S. Gundacker, M. Guthoff, R. Guida, J. Hammer, M. Hansen, P. Harris, J. Hegeman, V. Innocente, P. Janot, K. Kousouris, K. Krajczar, P. Lecoq, C. Lourenço, N. Magini, L. Malgeri, M. Mannelli, J. Marrouche, L. Masetti, F. Meijers, S. Mersi, E. Meschi, F. Moortgat, S. Morovic, M. Mulders, L. Orsini, L. Pape, E. Perez, A. Petrilli, G. Petrucciani, A. Pfeiffer, M. Pimiä, D. Piparo, M. Plagge, A. Racz, J. Rojo, G. Rolandi, M. Rovere, H. Sakulin, C. Schäfer, C. Schwick, A. Sharma, P. Siegrist, P. Silva, M. Simon, P. Sphicas, D. Spiga, J. Steggemann, B. Stieger, M. Stoye, Y. Takahashi, D. Treille, A. Tsirou, G. I. Veres, N. Wardle, H. K. Wöhri, H. Wollny, W. D. Zeuner, W. Bertl, K. Deiters, W. Erdmann, R. Horisberger, Q. Ingram, H. C. Kaestli, D. Kotlinski, U. Langenegger, D. Renker, T. Rohe, F. Bachmair, L. Bäni, L. Bianchini, M. A. Buchmann, B. Casal, N. Chanon, G. Dissertori, M. Dittmar, M. Donegà, M. Dünser, P. Eller, C. Grab, D. Hits, J. Hoss, W. Lustermann, B. Mangano, A. C. Marini, M. Marionneau, P. Martinez Ruiz del Arbol, M. Masciovecchio, D. Meister, N. Mohr, P. Musella, C. Nägeli, F. Nessi-Tedaldi, F. Pandolfi, F. Pauss, L. Perrozzi, M. Peruzzi, M. Quittnat, L. Rebane, M. Rossini, A. Starodumov, M. Takahashi, K. Theofilatos, R. Wallny, H. A. Weber, C. Amsler, M. F. Canelli, V. Chiochia, A. De Cosa, A. Hinzmann, T. Hreus, B. Kilminster, C. Lange, B. Millan Mejias, J. Ngadiuba, D. Pinna, P. Robmann, F. J. Ronga, S. Taroni, M. Verzetti, Y. Yang, M. Cardaci, K. H. Chen, C. Ferro, C. M. Kuo, W. Lin, Y. J. Lu, R. Volpe, S. S. Yu, P. Chang, Y. H. Chang, Y. Chao, K. F. Chen, P. H. Chen, C. Dietz, U. Grundler, W.-S. Hou, Y. F. Liu, R.-S. Lu, E. Petrakou, Y. M. Tzeng, R. Wilken, B. Asavapibhop, G. Singh, N. Srimanobhas, N. Suwonjandee, A. Adiguzel, M. N. Bakirci, S. Cerci, C. Dozen, I. Dumanoglu, E. Eskut, S. Girgis, G. Gokbulut, Y. Guler, E. Gurpinar, I. Hos, E. E. Kangal, A. Kayis Topaksu, G. Onengut, K. Ozdemir, S. Ozturk, A. Polatoz, D. Sunar Cerci, B. Tali, H. Topakli, M. Vergili, C. Zorbilmez, I. V. Akin, B. Bilin, S. Bilmis, H. Gamsizkan, B. Isildak, G. Karapinar, K. Ocalan, S. Sekmen, U. E. Surat, M. Yalvac, M. Zeyrek, E. A. Albayrak, E. Gülmez, M. Kaya, O. Kaya, T. Yetkin, K. Cankocak, F. I. Vardarlı, L. Levchuk, P. Sorokin, J. J. Brooke, E. Clement, D. Cussans, H. Flacher, J. Goldstein, M. Grimes, G. P. Heath, H. F. Heath, J. Jacob, L. Kreczko, C. Lucas, Z. Meng, D. M. Newbold, S. Paramesvaran, A. Poll, T. Sakuma, S. Seif El Nasr-storey, S. Senkin, V. J. Smith, T. Williams, K. W. Bell, A. Belyaev, C. Brew, R. M. Brown, D. J. A. Cockerill, J. A. Coughlan, K. Harder, S. Harper, E. Olaiya, D. Petyt, C. H. Shepherd-Themistocleous, A. Thea, I. R. Tomalin, T. Williams, W. J. Womersley, S. D. Worm, M. Baber, R. Bainbridge, O. Buchmuller, D. Burton, D. Colling, N. Cripps, P. Dauncey, G. Davies, M. Della Negra, P. Dunne, W. Ferguson, J. Fulcher, D. Futyan, G. Hall, G. Iles, M. Jarvis, G. Karapostoli, M. Kenzie, R. Lane, R. Lucas, L. Lyons, A.-M. Magnan, S. Malik, B. Mathias, J. Nash, A. Nikitenko, J. Pela, M. Pesaresi, K. Petridis, D. M. Raymond, S. Rogerson, A. Rose, C. Seez, P. Sharp, A. Tapper, M. Vazquez Acosta, T. Virdee, S. C. Zenz, J. E. Cole, P. R. Hobson, A. Khan, P. Kyberd, D. Leggat, D. Leslie, I. D. Reid, P. Symonds, L. Teodorescu, M. Turner, J. Dittmann, K. Hatakeyama, A. Kasmi, H. Liu, T. Scarborough, Z. Wu, O. Charaf, S. I. Cooper, C. Henderson, P. Rumerio, A. Avetisyan, T. Bose, C. Fantasia, P. Lawson, C. Richardson, J. Rohlf, J. St. John, L. Sulak, J. Alimena, E. Berry, S. Bhattacharya, G. Christopher, D. Cutts, Z. Demiragli, N. Dhingra, A. Ferapontov, A. Garabedian, U. Heintz, G. Kukartsev, E. Laird, G. Landsberg, M. Luk, M. Narain, M. Segala, T. Sinthuprasith, T. Speer, J. Swanson, R. Breedon, G. Breto, M. Calderon De La Barca Sanchez, S. Chauhan, M. Chertok, J. Conway, R. Conway, P. T. Cox, R. Erbacher, M. Gardner, W. Ko, R. Lander, M. Mulhearn, D. Pellett, J. Pilot, F. Ricci-Tam, S. Shalhout, J. Smith, M. Squires, D. Stolp, M. Tripathi, S. Wilbur, R. Yohay, R. Cousins, P. Everaerts, C. Farrell, J. Hauser, M. Ignatenko, G. Rakness, E. Takasugi, V. Valuev, M. Weber, K. Burt, R. Clare, J. Ellison, J. W. Gary, G. Hanson, J. Heilman, M. Ivova Rikova, P. Jandir, E. Kennedy, F. Lacroix, O. R. Long, A. Luthra, M. Malberti, M. Olmedo Negrete, A. Shrinivas, S. Sumowidagdo, S. Wimpenny, J. G. Branson, G. B. Cerati, S. Cittolin, R. T. D’Agnolo, A. Holzner, R. Kelley, D. Klein, J. Letts, I. Macneill, D. Olivito, S. Padhi, C. Palmer, M. Pieri, M. Sani, V. Sharma, S. Simon, M. Tadel, Y. Tu, A. Vartak, C. Welke, F. Würthwein, A. Yagil, D. Barge, J. Bradmiller-Feld, C. Campagnari, T. Danielson, A. Dishaw, V. Dutta, K. Flowers, M. Franco Sevilla, P. Geffert, C. George, F. Golf, L. Gouskos, J. Incandela, C. Justus, N. Mccoll, J. Richman, D. Stuart, W. To, C. West, J. Yoo, A. Apresyan, A. Bornheim, J. Bunn, Y. Chen, J. Duarte, A. Mott, H. B. Newman, C. Pena, M. Pierini, M. Spiropulu, J. R. Vlimant, R. Wilkinson, S. Xie, R. Y. Zhu, V. Azzolini, A. Calamba, B. Carlson, T. Ferguson, Y. Iiyama, M. Paulini, J. Russ, H. Vogel, I. Vorobiev, J. P. Cumalat, W. T. Ford, A. Gaz, M. Krohn, E. Luiggi Lopez, U. Nauenberg, J. G. Smith, K. Stenson, S. R. Wagner, J. Alexander, A. Chatterjee, J. Chaves, J. Chu, S. Dittmer, N. Eggert, N. Mirman, G. Nicolas Kaufman, J. R. Patterson, A. Ryd, E. Salvati, L. Skinnari, W. Sun, W. D. Teo, J. Thom, J. Thompson, J. Tucker, Y. Weng, L. Winstrom, P. Wittich, D. Winn, S. Abdullin, M. Albrow, J. Anderson, G. Apollinari, L. A. T. Bauerdick, A. Beretvas, J. Berryhill, P. C. Bhat, G. Bolla, K. Burkett, J. N. Butler, H. W. K. Cheung, F. Chlebana, S. Cihangir, V. D. Elvira, I. Fisk, J. Freeman, E. Gottschalk, L. Gray, D. Green, S. Grünendahl, O. Gutsche, J. Hanlon, D. Hare, R. M. Harris, J. Hirschauer, B. Hooberman, S. Jindariani, M. Johnson, U. Joshi, B. Klima, B. Kreis, S. Kwan, J. Linacre, D. Lincoln, R. Lipton, T. Liu, J. Lykken, K. Maeshima, J. M. Marraffino, V. I. Martinez Outschoorn, S. Maruyama, D. Mason, P. McBride, P. Merkel, K. Mishra, S. Mrenna, S. Nahn, C. Newman-Holmes, V. O’Dell, O. Prokofyev, E. Sexton-Kennedy, S. Sharma, A. Soha, W. J. Spalding, L. Spiegel, L. Taylor, S. Tkaczyk, N. V. Tran, L. Uplegger, E. W. Vaandering, R. Vidal, A. Whitbeck, J. Whitmore, F. Yang, D. Acosta, P. Avery, P. Bortignon, D. Bourilkov, M. Carver, D. Curry, S. Das, M. De Gruttola, G. P. Di Giovanni, R. D. Field, M. Fisher, I. K. Furic, J. Hugon, J. Konigsberg, A. Korytov, T. Kypreos, J. F. Low, K. Matchev, H. Mei, P. Milenovic, G. Mitselmakher, L. Muniz, A. Rinkevicius, L. Shchutska, M. Snowball, D. Sperka, J. Yelton, M. Zakaria, S. Hewamanage, S. Linn, P. Markowitz, G. Martinez, J. L. Rodriguez, T. Adams, A. Askew, J. Bochenek, B. Diamond, J. Haas, S. Hagopian, V. Hagopian, K. F. Johnson, H. Prosper, V. Veeraraghavan, M. Weinberg, M. M. Baarmand, M. Hohlmann, H. Kalakhety, F. Yumiceva, M. R. Adams, L. Apanasevich, D. Berry, R. R. Betts, I. Bucinskaite, R. Cavanaugh, O. Evdokimov, L. Gauthier, C. E. Gerber, D. J. Hofman, P. Kurt, C. O’Brien, I. D. Sandoval Gonzalez, C. Silkworth, P. Turner, N. Varelas, B. Bilki, W. Clarida, K. Dilsiz, M. Haytmyradov, J.-P. Merlo, H. Mermerkaya, A. Mestvirishvili, A. Moeller, J. Nachtman, H. Ogul, Y. Onel, F. Ozok, A. Penzo, R. Rahmat, S. Sen, P. Tan, E. Tiras, J. Wetzel, K. Yi, I. Anderson, B. A. Barnett, B. Blumenfeld, S. Bolognesi, D. Fehling, A. V. Gritsan, P. Maksimovic, C. Martin, M. Swartz, P. Baringer, A. Bean, G. Benelli, C. Bruner, J. Gray, R. P. Kenny, D. Majumder, M. Malek, M. Murray, D. Noonan, S. Sanders, J. Sekaric, R. Stringer, Q. Wang, J. S. Wood, I. Chakaberia, A. Ivanov, K. Kaadze, S. Khalil, M. Makouski, Y. Maravin, L. K. Saini, N. Skhirtladze, I. Svintradze, J. Gronberg, D. Lange, F. Rebassoo, D. Wright, A. Baden, A. Belloni, B. Calvert, S. C. Eno, J. A. Gomez, N. J. Hadley, R. G. Kellogg, T. Kolberg, Y. Lu, A. C. Mignerey, K. Pedro, A. Skuja, M. B. Tonjes, S. C. Tonwar, A. Apyan, R. Barbieri, W. Busza, I. A. Cali, M. Chan, L. Di Matteo, G. Gomez Ceballos, M. Goncharov, D. Gulhan, M. Klute, Y. S. Lai, Y.-J. Lee, A. Levin, P. D. Luckey, C. Paus, D. Ralph, C. Roland, G. Roland, G. S. F. Stephans, K. Sumorok, D. Velicanu, J. Veverka, B. Wyslouch, M. Yang, A. S. Yoon, M. Zanetti, V. Zhukova, B. Dahmes, A. De Benedetti, A. Gude, S. C. Kao, K. Klapoetke, Y. Kubota, J. Mans, S. Nourbakhsh, N. Pastika, R. Rusack, A. Singovsky, N. Tambe, J. Turkewitz, J. G. Acosta, L. M. Cremaldi, R. Kroeger, S. Oliveros, L. Perera, D. A. Sanders, D. Summers, E. Avdeeva, K. Bloom, S. Bose, D. R. Claes, A. Dominguez, R. Gonzalez Suarez, J. Keller, D. Knowlton, I. Kravchenko, J. Lazo-Flores, F. Meier, F. Ratnikov, G. R. Snow, M. Zvada, J. Dolen, A. Godshalk, I. Iashvili, S. Jain, A. Kharchilava, A. Kumar, S. Rappoccio, G. Alverson, E. Barberis, D. Baumgartel, M. Chasco, A. Massironi, D. Nash, T. Orimoto, D. Trocino, D. Wood, J. Zhang, A. Anastassov, K. A. Hahn, A. Kubik, L. Lusito, N. Mucia, N. Odell, B. Pollack, A. Pozdnyakov, M. Schmitt, S. Stoynev, K. Sung, M. Velasco, S. Won, A. Brinkerhoff, K. M. Chan, A. Drozdetskiy, M. Hildreth, C. Jessop, D. J. Karmgard, N. Kellams, K. Lannon, S. Lynch, N. Marinelli, Y. Musienko, T. Pearson, M. Planer, R. Ruchti, N. Valls, G. Smith, M. Wayne, M. Wolf, A. Woodard, L. Antonelli, J. Brinson, B. Bylsma, L. S. Durkin, S. Flowers, A. Hart, C. Hill, R. Hughes, K. Kotov, T. Y. Ling, W. Luo, D. Puigh, M. Rodenburg, B. L. Winer, H. Wolfe, H. W. Wulsin, O. Driga, P. Elmer, J. Hardenbrook, P. Hebda, S. A. Koay, P. Lujan, D. Marlow, T. Medvedeva, M. Mooney, J. Olsen, P. Piroué, X. Quan, H. Saka, D. Stickland, C. Tully, J. S. Werner, A. Zuranski, E. Brownson, S. Malik, H. Mendez, J. E. Ramirez Vargas, V. E. Barnes, D. Benedetti, D. Bortoletto, M. De Mattia, L. Gutay, Z. Hu, M. K. Jha, M. Jones, K. Jung, M. Kress, N. Leonardo, D. H. Miller, N. Neumeister, B. C. Radburn-Smith, X. Shi, I. Shipsey, D. Silvers, A. Svyatkovskiy, F. Wang, W. Xie, L. Xu, J. Zablocki, N. Parashar, J. Stupak, A. Adair, B. Akgun, K. M. Ecklund, F. J. M. Geurts, W. Li, B. Michlin, B. P. Padley, R. Redjimi, J. Roberts, J. Zabel, B. Betchart, A. Bodek, R. Covarelli, P. de Barbaro, R. Demina, Y. Eshaq, T. Ferbel, A. Garcia-Bellido, P. Goldenzweig, J. Han, A. Harel, O. Hindrichs, A. Khukhunaishvili, S. Korjenevski, G. Petrillo, D. Vishnevskiy, R. Ciesielski, L. Demortier, K. Goulianos, C. Mesropian, S. Arora, A. Barker, J. P. Chou, C. Contreras-Campana, E. Contreras-Campana, D. Duggan, D. Ferencek, Y. Gershtein, R. Gray, E. Halkiadakis, D. Hidas, S. Kaplan, A. Lath, S. Panwalkar, M. Park, R. Patel, S. Salur, S. Schnetzer, D. Sheffield, S. Somalwar, R. Stone, S. Thomas, P. Thomassen, M. Walker, K. Rose, S. Spanier, A. York, O. Bouhali, A. Castaneda Hernandez, R. Eusebi, W. Flanagan, J. Gilmore, T. Kamon, V. Khotilovich, V. Krutelyov, R. Montalvo, I. Osipenkov, Y. Pakhotin, A. Perloff, J. Roe, A. Rose, A. Safonov, I. Suarez, A. Tatarinov, K. A. Ulmer, N. Akchurin, C. Cowden, J. Damgov, C. Dragoiu, P. R. Dudero, J. Faulkner, K. Kovitanggoon, S. Kunori, S. W. Lee, T. Libeiro, I. Volobouev, E. Appelt, A. G. Delannoy, S. Greene, A. Gurrola, W. Johns, C. Maguire, Y. Mao, A. Melo, M. Sharma, P. Sheldon, B. Snook, S. Tuo, J. Velkovska, M. W. Arenton, S. Boutle, B. Cox, B. Francis, J. Goodell, R. Hirosky, A. Ledovskoy, H. Li, C. Lin, C. Neu, J. Wood, C. Clarke, R. Harr, P. E. Karchin, C. Kottachchi Kankanamge Don, P. Lamichhane, J. Sturdy, D. A. Belknap, D. Carlsmith, M. Cepeda, S. Dasu, L. Dodd, S. Duric, E. Friis, R. Hall-Wilton, M. Herndon, A. Hervé, P. Klabbers, A. Lanaro, C. Lazaridis, A. Levine, R. Loveless, A. Mohapatra, I. Ojalvo, T. Perry, G. A. Pierro, G. Polese, I. Ross, T. Sarangi, A. Savin, W. H. Smith, D. Taylor, C. Vuosalo, N. Woods, [Authorinst]The CMS Collaboration

**Affiliations:** Yerevan Physics Institute, Yerevan, Armenia; Institut für Hochenergiephysik der OeAW, Wien, Austria; National Centre for Particle and High Energy Physics, Minsk, Belarus; Universiteit Antwerpen, Antwerpen, Belgium; Vrije Universiteit Brussel, Brussel, Belgium; Université Libre de Bruxelles, Bruxelles, Belgium; Ghent University, Ghent, Belgium; Université Catholique de Louvain, Louvain-la-Neuve, Belgium; Université de Mons, Mons, Belgium; Centro Brasileiro de Pesquisas Fisicas, Rio de Janeiro, Brazil; Universidade do Estado do Rio de Janeiro, Rio de Janeiro, Brazil; Universidade Estadual Paulista, Universidade Federal do ABC, São Paulo, Brazil; Institute for Nuclear Research and Nuclear Energy, Sofia, Bulgaria; University of Sofia, Sofia, Bulgaria; Institute of High Energy Physics, Beijing, China; State Key Laboratory of Nuclear Physics and Technology, Peking University, Beijing, China; Universidad de Los Andes, Bogota, Colombia; University of Split, Faculty of Electrical Engineering, Mechanical Engineering and Naval Architecture, Split, Croatia; University of Split, Faculty of Science, Split, Croatia; Institute Rudjer Boskovic, Zagreb, Croatia; University of Cyprus, Nicosia, Cyprus; Charles University, Prague, Czech Republic; Academy of Scientific Research and Technology of the Arab Republic of Egypt, Egyptian Network of High Energy Physics, Cairo, Egypt; National Institute of Chemical Physics and Biophysics, Tallinn, Estonia; Department of Physics, University of Helsinki, Helsinki, Finland; Helsinki Institute of Physics, Helsinki, Finland; Lappeenranta University of Technology, Lappeenranta, Finland; DSM/IRFU, CEA/Saclay, Gif-sur-Yvette, France; Laboratoire Leprince-Ringuet, Ecole Polytechnique, IN2P3-CNRS, Palaiseau, France; Institut Pluridisciplinaire Hubert Curien, Université de Strasbourg, Université de Haute Alsace Mulhouse, CNRS/IN2P3, Strasbourg, France; Centre de Calcul de l’Institut National de Physique Nucleaire et de Physique des Particules, CNRS/IN2P3, Villeurbanne, France; Institut de Physique Nucléaire de Lyon, Université de Lyon, Université Claude Bernard Lyon 1, CNRS-IN2P3, Villeurbanne, France; Institute of High Energy Physics and Informatization, Tbilisi State University, Tbilisi, Georgia; RWTH Aachen University, I. Physikalisches Institut, Aachen, Germany; RWTH Aachen University, III. Physikalisches Institut A, Aachen, Germany; RWTH Aachen University, III. Physikalisches Institut B, Aachen, Germany; Deutsches Elektronen-Synchrotron, Hamburg, Germany; University of Hamburg, Hamburg, Germany; Institut für Experimentelle Kernphysik, Karlsruhe, Germany; Institute of Nuclear and Particle Physics (INPP), NCSR Demokritos, Aghia Paraskevi, Greece; University of Athens, Athens, Greece; University of Ioánnina, Ioánnina, Greece; Wigner Research Centre for Physics, Budapest, Hungary; Institute of Nuclear Research ATOMKI, Debrecen, Hungary; University of Debrecen, Debrecen, Hungary; National Institute of Science Education and Research, Bhubaneswar, India; Panjab University, Chandigarh, India; University of Delhi, Delhi, India; Saha Institute of Nuclear Physics, Kolkata, India; Bhabha Atomic Research Centre, Mumbai, India; Tata Institute of Fundamental Research, Mumbai, India; Institute for Research in Fundamental Sciences (IPM), Tehran, Iran; University College Dublin, Dublin, Ireland; INFN Sezione di Bari, Università di Bari, Politecnico di Bari, Bari, Italy; INFN Sezione di Bologna, Università di Bologna, Bologna, Italy; INFN Sezione di Catania, Università di Catania, CSFNSM, Catania, Italy; INFN Sezione di Firenze, Università di Firenze, Firenze, Italy; INFN Laboratori Nazionali di Frascati, Frascati, Italy; INFN Sezione di Genova, Università di Genova, Genoa, Italy; INFN Sezione di Milano-Bicocca, Università di Milano-Bicocca, Milan, Italy; INFN Sezione di Napoli, Università di Napoli ’Federico II’, Università della Basilicata (Potenza), Università G. Marconi (Roma), Naples, Italy; INFN Sezione di Padova, Università di Padova, Università di Trento (Trento), Padua, Italy; INFN Sezione di Pavia, Università di Pavia, Pavia, Italy; INFN Sezione di Perugia, Università di Perugia, Perugia, Italy; INFN Sezione di Pisa, Università di Pisa, Scuola Normale Superiore di Pisa, Pisa, Italy; INFN Sezione di Roma, Università di Roma, Rome, Italy; INFN Sezione di Torino, Università di Torino, Università del Piemonte Orientale (Novara), Turin, Italy; INFN Sezione di Trieste, Università di Trieste, Trieste, Italy; Kangwon National University, Chunchon, Korea; Kyungpook National University, Taegu, Korea; Chonbuk National University, Chonju, Korea; Chonnam National University, Institute for Universe and Elementary Particles, Kwangju, Korea; Korea University, Seoul, Korea; University of Seoul, Seoul, Korea; Sungkyunkwan University, Suwon, Korea; Vilnius University, Vilnius, Lithuania; National Centre for Particle Physics, Universiti Malaya, Kuala Lumpur, Malaysia; Centro de Investigacion y de Estudios Avanzados del IPN, Mexico City, Mexico; Universidad Iberoamericana, Mexico City, Mexico; Benemerita Universidad Autonoma de Puebla, Puebla, Mexico; Universidad Autónoma de San Luis Potosí, San Luis Potosí, Mexico; University of Auckland, Auckland, New Zealand; University of Canterbury, Christchurch, New Zealand; National Centre for Physics, Quaid-I-Azam University, Islamabad, Pakistan; National Centre for Nuclear Research, Swierk, Poland; Institute of Experimental Physics, Faculty of Physics, University of Warsaw, Warsaw, Poland; Laboratório de Instrumentação e Física Experimental de Partículas, Lisbon, Portugal; Joint Institute for Nuclear Research, Dubna, Russia; Petersburg Nuclear Physics Institute, Gatchina (St. Petersburg), Russia; Institute for Nuclear Research, Moscow, Russia; Institute for Theoretical and Experimental Physics, Moscow, Russia; P. N. Lebedev Physical Institute, Moscow, Russia; Skobeltsyn Institute of Nuclear Physics, Lomonosov Moscow State University, Moscow, Russia; State Research Center of Russian Federation, Institute for High Energy Physics, Protvino, Russia; University of Belgrade, Faculty of Physics and Vinca Institute of Nuclear Sciences, Belgrade, Serbia; Centro de Investigaciones Energéticas Medioambientales y Tecnológicas (CIEMAT), Madrid, Spain; Universidad Autónoma de Madrid, Madrid, Spain; Universidad de Oviedo, Oviedo, Spain; Instituto de Física de Cantabria (IFCA), CSIC-Universidad de Cantabria, Santander, Spain; CERN, European Organization for Nuclear Research, Geneva, Switzerland; Paul Scherrer Institut, Villigen, Switzerland; Institute for Particle Physics, ETH Zurich, Zurich, Switzerland; Universität Zürich, Zurich, Switzerland; National Central University, Chung-Li, Taiwan; National Taiwan University (NTU), Taipei, Taiwan; Faculty of Science, Department of Physics, Chulalongkorn University, Bangkok, Thailand; Cukurova University, Adana, Turkey; Physics Department, Middle East Technical University, Ankara, Turkey; Bogazici University, Istanbul, Turkey; Istanbul Technical University, Istanbul, Turkey; National Scientific Center, Kharkov Institute of Physics and Technology, Kharkiv, Ukraine; University of Bristol, Bristol, UK; Rutherford Appleton Laboratory, Didcot, UK; Imperial College, London, UK; Brunel University, Uxbridge, UK; Baylor University, Waco, USA; The University of Alabama, Tuscaloosa, USA; Boston University, Boston, USA; Brown University, Providence, USA; University of California, Davis, USA; University of California, Los Angeles, USA; University of California, Riverside, Riverside, USA; University of California, San Diego, La Jolla, USA; University of California, Santa Barbara, Santa Barbara, USA; California Institute of Technology, Pasadena, USA; Carnegie Mellon University, Pittsburgh, USA; University of Colorado at Boulder, Boulder, USA; Cornell University, Ithaca, USA; Fairfield University, Fairfield, USA; Fermi National Accelerator Laboratory, Batavia, USA; University of Florida, Gainesville, USA; Florida International University, Miami, USA; Florida State University, Tallahassee, USA; Florida Institute of Technology, Melbourne, USA; University of Illinois at Chicago (UIC), Chicago, USA; The University of Iowa, Iowa City, USA; Johns Hopkins University, Baltimore, USA; The University of Kansas, Lawrence, USA; Kansas State University, Manhattan, USA; Lawrence Livermore National Laboratory, Livermore, USA; University of Maryland, College Park, USA; Massachusetts Institute of Technology, Cambridge, USA; University of Minnesota, Minneapolis, USA; University of Mississippi, Oxford, USA; University of Nebraska-Lincoln, Lincoln, USA; State University of New York at Buffalo, Buffalo, USA; Northeastern University, Boston, USA; Northwestern University, Evanston, USA; University of Notre Dame, Notre Dame, USA; The Ohio State University, Columbus, USA; Princeton University, Princeton, USA; University of Puerto Rico, Mayaguez, USA; Purdue University, West Lafayette, USA; Purdue University Calumet, Hammond, USA; Rice University, Houston, USA; University of Rochester, Rochester, USA; The Rockefeller University, New York, USA; Rutgers, The State University of New Jersey, Piscataway, USA; University of Tennessee, Knoxville, USA; Texas A&M University, College Station, USA; Texas Tech University, Lubbock, USA; Vanderbilt University, Nashville, USA; University of Virginia, Charlottesville, USA; Wayne State University, Detroit, USA; University of Wisconsin, Madison, USA; CERN, Geneva, Switzerland; Seoul National University, Seoul, Korea

## Abstract

Measurements of the differential and double-differential Drell–Yan cross sections in the dielectron and dimuon channels are presented. They are based on proton–proton collision data at $\sqrt{s} = 8\,\text {TeV}$ recorded with the CMS detector at the LHC and corresponding to an integrated luminosity of 19.7$\,\text {fb}^{-1}$. The measured inclusive cross section in the $\mathrm{Z}$ peak region (60–120$\,\text {GeV}$), obtained from the combination of the dielectron and dimuon channels, is $1138 \pm 8\,\text {(exp)} \pm 25\,\text {(theo)} \pm 30\,\text {(lumi)} \text {\,pb}$, where the statistical uncertainty is negligible. The differential cross section $\mathrm{d}\sigma /\mathrm{d}{}m$ in the dilepton mass range 15–2000$\,\text {GeV}$ is measured and corrected to the full phase space. The double-differential cross section $\mathrm{d}^2\sigma /\mathrm{d}{}m\,\mathrm{d}|y |$ is also measured over the mass range 20 to 1500$\,\text {GeV}$ and absolute dilepton rapidity from 0 to 2.4. In addition, the ratios of the normalized differential cross sections measured at $\sqrt{s} = 7$ and 8$\,\text {TeV}$ are presented. These measurements are compared to the predictions of perturbative QCD at next-to-leading and next-to-next-to-leading (NNLO) orders using various sets of parton distribution functions (PDFs). The results agree with the NNLO theoretical predictions computed with fewz 3.1 using the CT10 NNLO and NNPDF2.1 NNLO PDFs. The measured double-differential cross section and ratio of normalized differential cross sections are sufficiently precise to constrain the proton PDFs.

## Introduction

At hadron colliders, Drell–Yan (DY) lepton pairs are produced via $\gamma ^{*}/\mathrm{Z}$ exchange in the $s$ channel. Theoretical calculations of the differential cross section $\mathrm{d}\sigma /\mathrm{d}{}m$ and the double-differential cross section $\mathrm{d}^2\sigma /\mathrm{d}{}m\,\mathrm{d}|y |$, where $m$ is the dilepton invariant mass and $|y |$ is the absolute value of the dilepton rapidity, are well established in the standard model (SM) up to the next-to-next-to-leading order (NNLO) in perturbative quantum chromodynamics (QCD) [1–4]. The rapidity distributions of the gauge bosons $\gamma ^{*}/\mathrm{Z}$ are sensitive to the parton content of the proton.

The rapidity and the invariant mass of the dilepton system produced in proton–proton collisions are related at leading order to the longitudinal momentum fractions $x_+$ and $x_-$ carried by the two interacting partons according to the formula $x_\pm =(m/\sqrt{s}) \mathrm{e}^{\pm y}$. Hence, the rapidity and mass distributions are sensitive to the parton distribution functions (PDFs) of the interacting partons. The differential cross sections are measured with respect to $|y |$ since the rapidity distribution is symmetric about zero. The high center-of-mass energy at the CERN LHC permits the study of DY production in regions of the Bjorken scaling variable and evolution scale $Q^2=x_+x_-s$ that were not accessible in previous experiments [5–10]. The present analysis covers the ranges $0.0003 < x_\pm < 1.0$ and $600 < Q^2 < 750{,}000\,\text {GeV} ^2$ in the double-differential cross section measurement. The differential cross section $\mathrm{d}\sigma /\mathrm{d}{}m$ is measured in an even wider range $300 < Q^2 < 3{,}000{,}000\,\text {GeV} ^2$.

The increase in the center-of-mass energy at the LHC from 7 to 8$\,\text {TeV}$ provides an opportunity to measure the ratios and double-differential ratios of cross sections of various hard processes, including the DY process. Measurements of the DY process in proton–proton collisions depend on various theoretical parameters such as the QCD running coupling constant, PDFs, and renormalization and factorization scales. The theoretical systematic uncertainties in the cross section measurements for a given process at different center-of-mass energies are substantial but correlated, so that the ratios of differential cross sections normalized to the $\mathrm{Z}$ boson production cross section (double ratios) can be measured very precisely [11].

This paper presents measurements of the DY differential cross section $$\mathrm{d}\sigma /\mathrm{d}{}m$$ in the mass range $$15 < m < 2000$$$$\,\text {GeV}$$, extending the measurement reported in [12], and of the double-differential cross section $$\mathrm{d}^2\sigma /\mathrm{d}{}m\,\mathrm{d}|y |$$ in the mass range $$20 < m < 1500$$$$\,\text {GeV}$$ and absolute dilepton rapidity from 0 to 2.4. In addition, the double ratios measured at 7 and 8$$\,\text {TeV}$$ are presented. The measurements are based on a data sample of proton–proton collisions with a center-of-mass energy $$\sqrt{s} = 8\,\text {TeV} $$, collected with the CMS detector and corresponding to an integrated luminosity of 19.7$$\,\text {fb}^{-1}$$. Integrated luminosities of 4.8$$\,\text {fb}^{-1}$$ (dielectron) and 4.5$$\,\text {fb}^{-1}$$ (dimuon) at $$\sqrt{s} = 7\,\text {TeV} $$ are used for the double ratio measurements.

Imperfect knowledge of PDFs [13, 14] is the dominant source of theoretical systematic uncertainties in the DY cross section predictions at low mass. The PDF uncertainty is larger than the achievable experimental precision, making the double-differential cross section and the double ratio measurements in bins of rapidity an effective input for PDF constraints. The inclusion of DY cross section and double ratio data in PDF fits is expected to provide substantial constraints for the strange quark and the light sea quark PDFs in the small Bjorken $x$ region ($0.001 < x < 0.1$).

The DY differential cross section has been measured by the CDF, D0, ATLAS, and CMS experiments [12, 15–19]. The current knowledge of the PDFs and the importance of the LHC measurements are reviewed in [20, 21]. Measuring the DY differential cross section $\mathrm{d}\sigma /\mathrm{d}{}m$ is important for various LHC physics analyses. DY events pose a major source of background for processes such as top quark pair production, diboson production, and Higgs measurements with lepton final states, as well as for searches for new physics beyond the SM, such as the production of high-mass dilepton resonances.

The differential cross sections are first measured separately for both lepton flavors and found to agree. The combined cross section measurement is then compared to the NNLO QCD predictions computed with fewz  3.1 [22] using the CT10 NNLO PDF. The $\mathrm{d}^2\sigma /\mathrm{d}{}m\,\mathrm{d}|y |$ measurement is compared to the NNLO theoretical predictions computed with fewz  3.1 using the CT10 and NNPDF2.1 NNLO PDFs [23, 24].

## CMS detector

The central feature of the CMS detector is a superconducting solenoid of 6$\text {\,m}$ internal diameter and 13$\text {\,m}$ length, providing a magnetic field of 3.8$\text {\,T}$. Within the field volume are a silicon tracker, a crystal electromagnetic calorimeter (ECAL), and a brass/scintillator hadron calorimeter (HCAL). The tracker is composed of a pixel detector and a silicon strip tracker, which are used to measure charged-particle trajectories and cover the full azimuthal angle and the pseudorapidity interval $|\eta | < 2.5$.

Muons are detected with four planes of gas-ionization detectors. These muon detectors are installed outside the solenoid and sandwiched between steel layers, which serve both as hadron absorbers and as a return yoke for the magnetic field flux. They are made using three technologies: drift tubes, cathode strip chambers, and resistive-plate chambers. Muons are measured in the pseudorapidity window $|\eta | < 2.4$. Electrons are detected using the energy deposition in the ECAL, which consists of nearly 76,000 lead tungstate crystals that are distributed in the barrel region ($|\eta | < 1.479$) and two endcap ($1.479 < |\eta | < 3$) regions.

The CMS experiment uses a two-level trigger system. The level-1 trigger, composed of custom processing hardware, selects events of interest at an output rate of 100$\text {\,kHz}$ using information from the calorimeters and muon detectors [25]. The high-level trigger (HLT) is software based and further decreases the event collection rate to a few hundred hertz by using the full event information, including that from the tracker [26]. A more detailed description of the CMS detector, together with a definition of the coordinate system used and the relevant kinematic variables, can be found in [27].

## Simulated samples

Several simulated samples are used for determining efficiencies, acceptances, and backgrounds from processes that result in two leptons, and for the determination of systematic uncertainties. The DY signal samples with $\mathrm {e}^{+}\mathrm {e}^{-}$ and $\mathrm {\mu ^{+}}\mathrm {\mu ^{-}}$ final states are generated with the next-to-leading (NLO) generator powheg  [28–31] interfaced with the pythia v6.4.24 [32] parton shower generator. pythia is used to model QED final-state radiation (FSR).

The powheg simulated sample is based on NLO calculations, and a correction is applied to take into account higher-order QCD and electroweak (EW) effects. The correction factors binned in dilepton rapidity $y$ and transverse momentum $p_{\mathrm {T}}$ are determined in each invariant-mass bin to be the ratio of the double-differential cross sections calculated at NNLO QCD and NLO EW with fewz  3.1 and at NLO with powheg, as described in [12]. The corresponding higher-order effects depend on the dilepton kinematic variables. Higher-order EW corrections are small in comparison to FSR corrections. They increase for invariant masses in the $\text {TeV}$ region [33], but are insignificant compared to the experimental precision for the whole mass range under study. The NNLO QCD effects are most important in the low-mass region. The effect of the correction factors on the acceptance ranges up to 50 % in the low-mass region (below 40$\,\text {GeV}$), but is almost negligible in the high-mass region (above 200$\,\text {GeV}$).

The main SM background processes are simulated with powheg ($\mathrm {DY}\rightarrow {\tau }^{+}{\tau }^{-}$, single top quark) and with MadGraph  [34] ($\mathrm{t}\overline{\mathrm{t}}$, diboson events $\mathrm {W}\mathrm {W}/\mathrm {W}\mathrm{Z}/\mathrm{Z}\mathrm{Z}$). Both powheg and MadGraph are interfaced with the tauola package [35], which handles decays of $\tau$ leptons. The normalization of the $\mathrm{t}\overline{\mathrm{t}}$ sample is set to the NNLO cross section of 245.8$\text {\,pb}$  [36]. Multijet QCD background events are produced with pythia.

All generated events are processed through a detailed simulation of the CMS detector based on Geant4  [37] and are reconstructed using the same algorithms used for the data. The proton structure is defined using the CT10 [23] PDFs. The simulation includes the effects of multiple interactions per bunch crossing [38] (pileup) with the simulated distribution of the number of interactions per LHC beam crossing corrected to match that observed in data.

## Object reconstruction and event selection

The events used in the analysis are selected with a dielectron or a dimuon trigger. Dielectron events are triggered by the presence of two electron candidates that pass loose requirements on the electron quality and isolation with a minimum transverse momentum $p_{\mathrm {T}}$ of 17$\,\text {GeV}$ for one of the electrons and 8$\,\text {GeV}$ for the other. The dimuon trigger requires one muon with $p_{\mathrm {T}} > 17\,\text {GeV}$ and a second muon with $p_{\mathrm {T}} > 8\,\text {GeV}$.

The offline reconstruction of the electrons begins with the clustering of energy depositions in the ECAL. The energy clusters are then matched to the electron tracks. Electrons are identified by means of shower shape variables. Each electron is required to be consistent with originating from the primary vertex in the event. Energetic photons produced in a $\mathrm {p}\mathrm {p}$ collision may interact with the detector material and convert into an electron–positron pair. The electrons or positrons originating from such photon conversions are suppressed by requiring that there be no more than one missing tracker hit between the primary vertex and the first hit on the reconstructed track matched to the electron; candidates are also rejected if they form a pair with a nearby track that is consistent with a conversion. Additional details on electron reconstruction and identification can be found in [39–42]. No charge requirements are imposed on the electron pairs to avoid inefficiency due to nonnegligible charge misidentification.

At the offline muon reconstruction stage, the data from the muon detectors are matched and fitted to data from the silicon tracker to form muon candidates. The muon candidates are required to pass the standard CMS muon identification and track quality criteria [43]. To suppress the background contributions due to muons originating from heavy-quark decays and nonprompt muons from hadron decays, both muons are required to be isolated from other particles. Requirements on the impact parameter and the opening angle between the two muons are further imposed to reject cosmic ray muons. In order to reject muons from light-meson decays, a common vertex for the two muons is required. More details on muon reconstruction and identification can be found in [12] and [43]. Events are selected for further analysis if they contain oppositely charged muon pairs meeting the above requirements. The candidate with the highest $\chi ^2$ probability from a kinematic fit to the dimuon vertex is selected.

Electron and muon isolation criteria are based on measuring the sum of energy depositions associated with photons and charged and neutral hadrons reconstructed and identified by means of the CMS particle-flow algorithm [44–47]. Isolation sums are evaluated in a circular region of the ($\eta$,$\phi$) plane around the lepton candidate with $\Delta R < 0.3$ (where $\Delta R = \sqrt{{(\Delta \eta )^2+(\Delta \phi )^2}}$), and are corrected for the contribution from pileup.

Each lepton is required to be within the geometrical acceptance of $|\eta | < 2.4$. The leading lepton in the event is required to have $p_{\mathrm {T}} > 20\,\text {GeV}$ and the trailing lepton $p_{\mathrm {T}} > 10\,\text {GeV}$, which corresponds to the plateau of the trigger efficiency. Both lepton candidates in each event used in the offline analysis are required to match HLT trigger objects.

After event selection, the analysis follows a series of steps. First, backgrounds are estimated. Next, the observed background-subtracted yield is unfolded to correct for the effects of the migration of events among bins of mass and rapidity due to the detector resolution. The acceptance and efficiency corrections are then applied. Finally, the migration of events due to FSR is corrected. Systematic uncertainties associated with each of the analysis steps are evaluated.

## Background estimation

The major background contributions in the dielectron channel arise from ${\tau }^{+}{\tau }^{-}$ and $\mathrm{t}\overline{\mathrm{t}}$ processes in the low-mass region and from QCD events with multiple jets at high invariant mass. The background composition is somewhat different in the dimuon final state. Multijet events and DY production of ${\tau }^{+}{\tau }^{-}$ pairs are the dominant sources of background in the dimuon channel at low invariant mass and in the region just below the $\mathrm{Z}$ peak. Diboson and $\mathrm{t}\overline{\mathrm{t}}$ production followed by leptonic decays are the dominant sources of background at high invariant mass. Lepton pair production in $\gamma \gamma$-initiated processes, where both initial-state protons radiate a photon, is significant at high mass. The contribution from this channel is treated as an irreducible background and is estimated with fewz  3.1 [48]. To correct for this background, a bin-by-bin ratio of the DY cross sections with and without the photon-induced contribution is calculated. This bin-by-bin correction is applied after the mass resolution unfolding step, whereas corrections for other background for which we have simulated events are corrected before. This background correction is negligible at low mass and in the $\mathrm{Z}$ peak region, rising to approximately 20 % in the highest mass bin.

In the dielectron channel, the QCD multijet background is estimated with a data sample collected with the trigger requirement of a single electromagnetic cluster in the event. Non-QCD events, such as DY, are removed from the data sample using event selection and event subtraction based on simulation, leaving a sample of QCD events with characteristics similar to those in the analysis data sample. This sample is used to estimate the probability for a jet to pass the requirements of the electromagnetic trigger and to be falsely reconstructed as an electron. This probability is then applied to a sample of events with one electron and one jet to estimate the background contribution from an electron and a jet passing electron selection requirements. As the contribution from two jets passing the electron selections is considered twice in the previous method, the contribution from a sample with two jets multiplied by the square of the probability for jets passing the electron selection requirements is further subtracted.

The QCD multijet background in the dimuon channel is evaluated by selecting a control data sample before the isolation and charge sign requirements are applied, following the method described in [49].

The largest background consists of final states with particles decaying by EW interaction, producing electron or muon pairs, for example, $\mathrm{t}\overline{\mathrm{t}}$, ${\tau }^{+}{\tau }^{-}$, and $\mathrm {W}\mathrm {W}$. Notably, these final states contain electron–muon pairs at twice the rate of electron or muon pairs. These electron–muon pairs can be cleanly identified from a data sample of $\mathrm {e}\mathrm {\mu }$ events and properly scaled (taking into account the detector acceptance and efficiency) in order to calculate the background contribution to the dielectron and dimuon channels.

Background yields estimated from an $\mathrm {e}\mathrm {\mu }$ data sample are used to reduce the systematic uncertainty due to the limited theoretical knowledge of the cross sections of the SM processes. The residual differences between background contributions estimated from data and simulation are taken into account in the systematic uncertainty assignment, as detailed in Sect. 9.

**Fig. 1:**
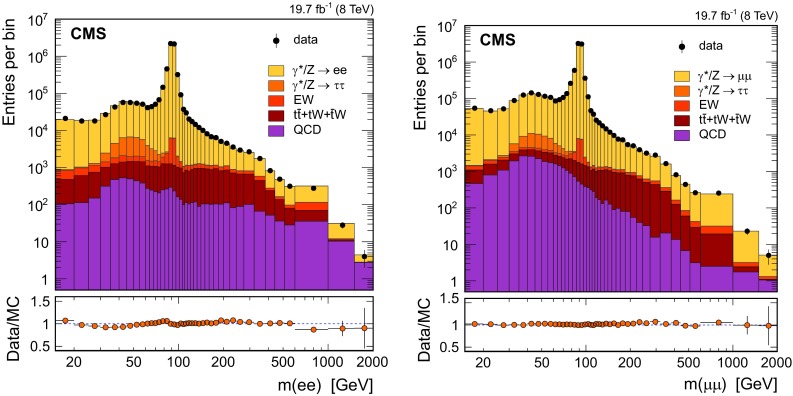
The dielectron (*left*) and dimuon (*right*) invariant-mass spectra observed in data and predicted by Monte Carlo (MC) simulation and the corresponding ratios of observed to expected yields. The QCD multijet contributions in both decay channels are predicted using control samples in data. The EW histogram indicates the diboson and $\mathrm {W}$+jets production. The simulated signal distributions are based on the NNLO-reweighted powheg sample. No other corrections are applied. *Error bars* are statistical only

The dilepton yields for data and simulated events in bins of invariant mass are reported in Fig. 1. The photon-induced background is absorbed in the signal distribution so no correction is applied at this stage. As shown in the figure, the background contribution at low mass is no larger than 5 % in both decay channels. In the high-mass region, background contamination is more significant, reaching approximately 50 % (30 %) in the dielectron (dimuon) distribution.

## Resolution and scale corrections

Imperfect lepton energy and momentum measurements can affect the reconstructed dilepton invariant-mass distributions. Correcting for these effects is important in precise measurements of differential cross sections.

A momentum scale correction to remove a bias in the reconstructed muon momenta due to the differences in the tracker misalignment between data and simulation and the residual magnetic field mismodeling is applied following the standard CMS procedure described in [50].

The electron energy deposits as measured in the ECAL are subject to a set of corrections involving information both from the ECAL and the tracker, following the standard CMS procedures for the 8$\,\text {TeV}$ data set [51]. A final electron energy scale correction, which goes beyond the standard set of corrections, is derived from an analysis of the $\mathrm{Z}\rightarrow \mathrm {e}^{+}\mathrm {e}^{-}$ peak according to the procedure described in [49], and consists of a simple factor of 1.001 applied to the electron energies to account for the different selection used in this analysis.

The detector resolution effects that cause a migration of events among the analysis bins are corrected through an iterative unfolding procedure [52]. This procedure maps the measured lepton distribution onto the true one, while taking into account the migration of events in and out of the mass and rapidity range of this measurement.

The effects of the unfolding correction in the differential cross section measurement are approximately 50 (20) % for dielectron (dimuon) channel in the $\mathrm{Z}$ peak region, where the invariant-mass spectrum changes steeply. Less significant effects, of the order of 15 % (5 %) in dielectron (dimuon) channel, are observed in other regions. The effect on the double-differential cross section measurement is less significant as both the invariant mass and rapidity bins are significantly wider than the respective detector resolutions. The effect for dielectrons reaches 15 % in the 45–60$\,\text {GeV}$ mass region and 5 % at high mass; it is, however, less than 1 % for dimuons over the entire invariant mass-rapidity range of study.

## Acceptance and efficiency

The acceptance $A$ is defined as the fraction of simulated signal events with both leptons passing the nominal $p_{\mathrm {T}}$ and $\eta$ requirements of the analysis. It is determined using the NNLO reweighted powheg simulated sample, after the simulation of FSR.

**Fig. 2:**
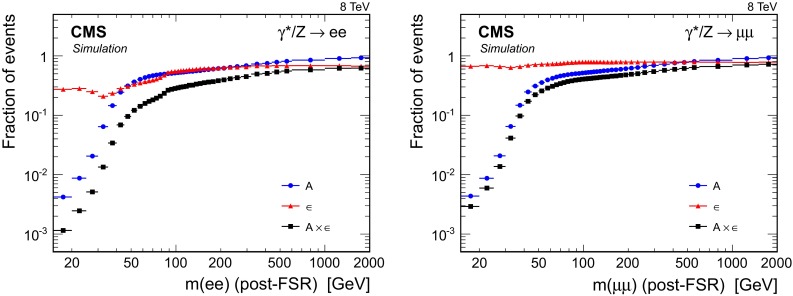
The DY acceptance, efficiency, and their product per invariant-mass bin in the dielectron channel (*top*) and the dimuon channel (*bottom*), where “post-FSR” means dilepton invariant mass after the simulation of FSR

The efficiency $\epsilon$ is the fraction of events in the DY simulated sample that are inside the acceptance and pass the full selection. The following equation holds:

1 $$\begin{aligned} A \epsilon \equiv { \frac{N^A}{N^\text {gen}}} { \frac{N^{\epsilon }}{N^A} } = {\frac{N^{\epsilon }}{N^\text {gen}}}, \end{aligned}$$

where $N^\text {gen}$ is the number of generated signal events in a given invariant-mass bin, $N^A$ is the number of events inside the geometrical and kinematic acceptances, and $N^{\epsilon }$ is the number of events passing the event selection criteria. Figure 2 shows the acceptance, the efficiency, and their product as functions of the dilepton invariant mass.

The DY acceptance is obtained from simulation. In the lowest mass bin it is only about 0.5 %, rapidly increasing to 50 % in the $\mathrm{Z}$ peak region and reaching over 90 % at high mass.

The efficiency is factorized into the reconstruction, identification, and isolation efficiencies and the event trigger efficiency. The factorization procedure takes into account the asymmetric $p_{\mathrm {T}}$ selections for the two legs of the dielectron trigger. The efficiency is obtained from simulation, rescaled with a correction factor that takes into account differences between data and simulation. The efficiency correction factor is determined in bins of lepton $p_{\mathrm {T}}$ and $\eta$ using $\mathrm{Z}\rightarrow \mathrm {e}^{+}\mathrm {e}^{-} (\mathrm {\mu ^{+}}\mathrm {\mu ^{-}})$ events in data and simulation with the tag-and-probe method [49] and is then applied as a weight to simulated events on a per-lepton basis.

A typical dimuon event efficiency is 70–80 % throughout the entire mass range. In the dielectron channel, the efficiency at low mass is only 20–40 % because of tighter lepton identification requirements, and reaches 65 % at high mass. The trigger efficiency for events within the geometrical acceptance is greater than 98 % (93 %) for the dielectron (dimuon) signal. The efficiency is significantly affected by the pileup in the event. The effect on the isolation efficiency is up to 5 % (about 1 %) in the dielectron (dimuon) channel.

A dip in the event efficiency in the mass range 30–40$\,\text {GeV}$, visible in Fig. 2, is caused by the combination of two factors. On one hand, the lepton reconstruction and identification efficiencies decrease as the lepton $p_{\mathrm {T}}$ decreases. On the other hand, the kinematic acceptance requirements preferentially select DY events produced beyond the leading order, which results in higher $p_{\mathrm {T}}$ leptons with higher reconstruction and identification efficiencies, in the mass range below 30–40$\,\text {GeV}$. The effect is more pronounced for dielectrons than for dimuons because the electron reconstruction and identification efficiencies depend more strongly on $p_{\mathrm {T}}$.

For the dimuon channel the efficiency correction factor is 0.95–1.10, rising up to 1.10 at high dimuon rapidity and falling to 0.95 at low mass. At low mass, the correction to the muon reconstruction and identification efficiency is dominant, falling to 0.94. In the dielectron channel, the efficiency correction factor is 0.96–1.05 in the $\mathrm{Z}$ peak region, and 0.90 at low mass. The correction factor rises to 1.05 at high dielectron rapidity. The correction to the electron identification and isolation efficiency is dominant in the dielectron channel, reaching 0.93 at low mass and 1.04 at high rapidity.

## Final-state QED radiation effects

The effect of photon radiation from the final-state leptons (FSR effect) moves the measured invariant mass of the dilepton pair to lower values, significantly affecting the mass spectrum, particularly in the region below the $\mathrm{Z}$ peak. A correction for FSR is performed to facilitate the comparison to the theoretical predictions and to properly combine the measurements in the dielectron and dimuon channels. The FSR correction is estimated separately from the detector resolution correction by means of the same unfolding technique. An additional bin-by-bin correction is applied for the events in which the leptons generated before FSR modeling (pre-FSR) fail the acceptance requirements, while they pass after the FSR modeling (post-FSR), following the approach described in [12]. The correction for the events not included in the response matrix is significant at low mass, reaching a maximum of 20 % in the lowest mass bin and decreasing to negligible levels in the $\mathrm{Z}$ peak region.

The magnitude of the FSR correction below the $\mathrm{Z}$ peak is on the order of 40–60 % (30–50 %) for the dielectron (dimuon) channel. In other mass regions, the effect is only 10–15 % in both channels. In the double-differential cross section measurement, the effect of FSR unfolding is not significant, typically a few percent, due to a larger mass bin size.

In order to compare the measurements corrected for FSR obtained in analyses with various event generators, the “dressed” lepton quantities can be considered. The dressed lepton four-momentum is defined as

2 $$\begin{aligned} \mathbf {p}^{\text {dressed}}_\ell = \mathbf {p}^{\text {post-FSR}}_\ell + \sum \mathbf {p}_{\gamma }, \end{aligned}$$

where all the simulated photons originating from leptons are summed within a cone of $\Delta R < 0.1$.

The correction to the cross sections from the post-FSR to the dressed level reaches a factor of 1.8 (1.3) in the dielectron (dimuon) channel immediately below the $\mathrm{Z}$ peak; it is around 0.8 in the low-mass region in both decay channels, and is close to 1.0 at high mass.

## Systematic uncertainties

*Acceptance uncertainty* The dominant uncertainty sources pertaining to the acceptance are (1) the theoretical uncertainty from imperfect knowledge of the nonperturbative PDFs contributing to the hard scattering and (2) the modeling uncertainty. The latter comes from the procedure to apply weights to the NLO simulated sample in order to reproduce NNLO kinematics and affects mostly the acceptance calculations at very low invariant mass. The PDF uncertainties for the differential and double-differential cross section measurements are calculated using the LHAGLUE interface to the PDF library LHAPDF 5.8.7 [53, 54] by applying a reweighting technique with asymmetric uncertainties as described in [55]. These contributions are largest at low and high masses (4–5 %) and decrease to less than 1 % for masses at the $\mathrm{Z}$ peak.

*Efficiency uncertainty* The systematic uncertainty in the efficiency estimation consists of two components: the uncertainty in the efficiency correction factor estimation and the uncertainty related to the number of simulated events. The efficiency correction factor reflects systematic deviations between data and simulation. It varies up to 10 % (7 %) for the dielectron (dimuon) channel. As discussed in Sect. 7, single-lepton efficiencies of several types are measured with the tag-and-probe procedure and are combined into efficiency correction factors. The tag-and-probe procedure provides the efficiencies for each lepton type and the associated statistical uncertainties. A variety of possible systematic biases in the tag-and-probe procedure have been taken into account, such as dependence on the binning in single-lepton $p_{\mathrm {T}}$ and $\eta$, dependence on the assumed shape of signal and background in the fit model, and the effect of pileup. In the dielectron channel, this uncertainty is as large as 3.2 % at low mass, and 6 % at high rapidity in the 200–1500$\,\text {GeV}$ region. The uncertainty in the dimuon channel is about 1 % in most of the analysis bins, reaching up to 4 % at high rapidity in the 200–1500$\,\text {GeV}$ mass region. The contribution from the dimuon vertex selection is small because its efficiency correction factor is consistent with being constant.

*Electron energy scale* In the dielectron channel, one of the leading systematic uncertainties is associated with the energy scale corrections for individual electrons. The corrections affect both the placement of a given candidate in a particular invariant-mass bin and the likelihood of surviving the kinematic selection. The energy scale corrections are calibrated to a precision of 0.1–0.2 %. The systematic uncertainties in the measured cross sections are estimated by varying the electron energy scale by 0.2 %. The uncertainty is relatively small at low masses. It reaches up to 6.2 % in the $\mathrm{Z}$ peak region where the mass bins are the narrowest and the variation of the cross section with mass is the largest.

*Muon momentum scale* The uncertainty in the muon momentum scale causes uncertainties in the efficiency estimation and background subtraction and affects the detector resolution unfolding. The muon momentum scale is calibrated to 0.02 % precision. The systematic uncertainty in the measured cross sections is determined by varying the muon momentum scale within its uncertainty. The largest effect on the final results is observed in the detector resolution unfolding step, reaching 2 %.

*Detector resolution* For both channels, the simulation of the CMS detector, used for detector resolution unfolding, provides a reliable description of the data. Possible small systematic errors in the unfolding are related to effects such as differences in the electron energy scale and muon momentum scale and uncertainties in FSR simulation and in simulated pileup. The impact of each of these effects on the measurements is studied separately, as described in this section. The detector resolution unfolding procedure itself has been thoroughly validated, including a variety of closure tests and comparisons between different event generators; the systematic uncertainty assigned to the unfolding procedure is based on the finite size of the simulated samples and a contribution due to the systematic difference in data and simulation. The latter must be taken into account because the response matrix is determined from simulation.

*Background uncertainty* The background estimation uncertainties are evaluated in the same way in both the dielectron and dimuon channels. The uncertainty in the background is comprised of the Poissonian statistical uncertainty of predicted backgrounds and the difference between the predictions from the data and simulation. The two components are combined in quadrature. The uncertainty in the background is no larger than 3.0 % (1.0 %) at low mass, reaching 16.3 % (4.6 %) in the highest mass bin in the dielectron (dimuon) channel.

$\gamma \gamma$*-initiated background uncertainty* The uncertainty in the correction for $\gamma \gamma$-initiated processes is estimated using fewz  3.1 with the NNPDF2.3QED PDF and consists of the statistical and PDF uncertainty contributions combined in quadrature.

*FSR simulation* The systematic uncertainty due to the model-dependent FSR simulation is estimated using two reweighting techniques described in [12] with the same procedure in both decay channels. The systematic uncertainty from modeling the FSR effects is as large as 2.5 % (1.1 %) in the dielectron (dimuon) channel in the 45–60$\,\text {GeV}$ region. The systematic uncertainties related to the FSR simulation in the electron channel primarily affect the detector resolution unfolding procedure. The impact of these uncertainties is greater for the electron channel than for the muon channel because of the partial recovery of FSR photons during the clustering of electron energy in the ECAL. The effect of the FSR simulation on other analysis steps for the electron channel is negligible in comparison to other systematic effects associated with those steps.

*Luminosity uncertainty* The uncertainty in the integrated luminosity recorded by CMS in the 2012 data set is 2.6 % [56].

Table 1 summarizes the systematic uncertainties for the dielectron and dimuon channels.

**Table 1:** Typical systematic uncertainties (in percent) at low mass (below 40$\,\text {GeV}$), in the $\mathrm{Z}$ peak region ($60 < m < 120$ $\,\text {GeV}$), and at high mass (above 200$\,\text {GeV}$) for the dielectron and dimuon channels; “—” means that the source does not apply

Sources	$\mathrm {e}^{+}\mathrm {e}^{-}$	$\mathrm {\mu ^{+}}\mathrm {\mu ^{-}}$
Efficiency	2.9, 0.5, 0.7	1.0, 0.4, 1.8
Detector resolution	1.2, 5.4, 1.8	0.6, 1.8, 1.6
Background estimation	2.2, 0.1, 13.8	1.0, 0.1, 4.6
Electron energy scale	0.2, 2.4, 2.0	–
Muon momentum scale	–	0.2, 1.7, 1.6
FSR simulation	0.4, 0.3, 0.3	0.4, 0.2, 0.5
Total experimental	3.7, 2.5, 14.0	1.6, 2.5, 5.4
Theoretical uncertainty	4.2, 1.6, 5.3	4.1, 1.6, 5.3
Luminosity	2.6, 2.6, 2.6	2.6, 2.6, 2.6
Total	6.3, 6.7, 15.3	5.1, 3.9, 8.0

*Systematic uncertainties in the double ratio* In the double ratio measurements most of the theoretical uncertainties are reduced. The PDF and modeling uncertainties in the acceptance and the systematic uncertainty in the FSR modeling are fully correlated between 7 and 8$\,\text {TeV}$ measurements. The relative uncertainty $\delta \sigma _{s_{i}}/\sigma _{s_{i}}$ in the cross section ratio corresponding to a correlated systematic source of uncertainty $s_{i}$ is estimated according to

3 $$\begin{aligned} \frac{\delta \sigma _{s_{i}}}{\sigma _{s_{i}}} = \frac{1+\delta _{s_{i}}(8\,\text {TeV})}{1+\delta _{s_{i}}(7\,\text {TeV})} - 1, \end{aligned}$$

where the $\delta _{s_{i}}$ are relative uncertainties caused by a source $s_{i}$ in the cross section measurements at $\sqrt{s} = 7$ and $8\,\text {TeV}$, respectively.

The systematic uncertainties that are considered uncorrelated between the two center-of-mass energies, including the uncertainties in efficiency correction estimation, background estimation, energy scale correction, unfolding, and integrated luminosity, are combined in quadrature.

## Results and discussion

The cross section measurements are first performed separately in the dielectron and dimuon decay channels and then combined using the procedure described in [57]. To assess the sensitivity of the measurement to PDF uncertainties, a comparison to theoretical calculations is performed using fewz  3.1 with CT10 and NNPDF2.1 NNLO PDFs [23, 24]. While the theory predictions are presented for NNPDF2.1, similar results are expected from the use of the more recent NNPDF3.0 [58].

### Differential cross section $\mathrm{d}\sigma /\mathrm{d}{}m$ measurement

The pre-FSR cross section in the full phase space is calculated as

4 $$\begin{aligned} \sigma ^{i} = \frac{N_\mathrm {u}^i}{A^i\epsilon ^iL_\text {int}}, \end{aligned}$$

where $N_\mathrm {u}^{i}$ is the number of events after background subtraction and unfolding procedures for detector resolution and FSR, $A^{i}$ is the acceptance, and $\epsilon ^{i}$ is the efficiency in a given invariant-mass bin $i$; $L_\text {int}$ is the total integrated luminosity.

The cross section in the $\mathrm{Z}$ peak region is calculated with Eq. (4) considering the mass region $60 < m < 120\,\text {GeV}$.

The $\mathrm{Z}$ peak cross section measurements in the dielectron and dimuon channels are summarized in Table 2.

**Table 2:** Absolute cross section measurements in the $\mathrm{Z}$ peak region ($60 < m < 120\,\text {GeV}$). The uncertainties in the measurements include the experimental and theoretical systematic sources and the uncertainty in the integrated luminosity. The statistical component is negligible

Channel	Cross section
Dielectron	$1141 \pm 11\,\text {(exp)} \pm 25\,\text {(theo)} \pm 30\,\text {(lumi)}$ $\text {\,pb}$
Dimuon	$1135 \pm 11\,\text {(exp)} \pm 25\,\text {(theo)} \pm 30\,\text {(lumi)}$ $\text {\,pb}$
Combined	$1138 \pm 8\,\text {(exp)} \pm 25\,\text {(theo)} \pm 30\,\text {(lumi)}$ $\text {\,pb}$

The measurements agree with NNLO theoretical predictions for the full phase space (i.e., $$1137 \pm 36$$$$\text {\,pb}$$, as calculated with fewz  3.1 and CT10 NNLO PDFs), and also with the previous CMS measurement [38].

The pre-FSR cross section for the full phase space is calculated in mass bins covering the range 15 to 2000$\,\text {GeV}$ by means of Eq. (4). The results are divided by the invariant-mass bin widths $\Delta m^i$.

The consistency of the differential cross section measurements obtained in the dielectron and dimuon channels is characterized by a $\chi ^2$ probability of 82 %, calculated from the total uncertainties. Therefore the measurements in the two channels are in agreement and are combined using the procedure defined in [57]. Based on the results in the two channels and their symmetric and positive definite covariance matrices, the estimates of the true cross section values are found as unbiased linear combinations of the input measurements having a minimum variance [59]. The uncertainties are considered to be uncorrelated between the two channels, with the exception of modeling, PDF, and luminosity uncertainties. The effects of correlations between the analysis bins and different systematic sources are taken into account in the combination procedure when constructing the covariance matrix.

**Fig. 3:**
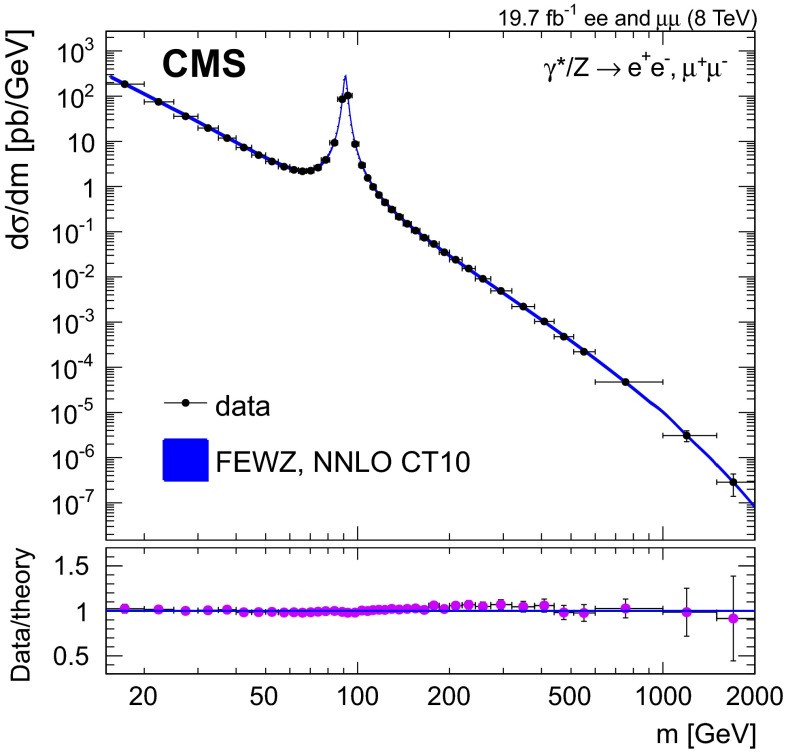
The DY differential cross section as measured in the combined dilepton channel and as predicted by NNLO fewz  3.1 with CT10 PDF calculations, for the full phase space. The data point abscissas are computed according to Eq. (6) in [60]. The $\chi ^2$ probability characterizing the consistency of the predicted and measured cross sections is 91 % with 41 degrees of freedom, calculated with total uncertainties while taking into account the correlated errors in the two channels

The result of the DY cross section measurement in the combined channel is presented in Fig. 3.

The theoretical prediction makes use of the fixed-order NNLO QCD calculation and the NLO EW correction to DY production initiated by purely weak processes. The $G_{\mu }$ input scheme [33] is used to fix the EW parameters in the model. The full spin correlations as well as the $\gamma ^{*}/\mathrm{Z}$ interference effects are included in the calculation. The combined measurement is in agreement with the NNLO theoretical predictions computed with fewz  3.1 using CT10 NNLO. The uncertainty band in Fig. 3 for the theoretical calculation represents the combination in quadrature of the statistical uncertainty from the fewz  3.1 calculation and the 68 % confidence level (CL) uncertainty from the PDFs. The uncertainties related to QCD evolution scale dependence are evaluated by varying the renormalization and factorization scales simultaneously between the values 2$m$, $m$, and $m$/2, with $m$ corresponding to the middle of the invariant mass bin. The scale variation uncertainties reach up to 2 % and are included in the theoretical error band.

### Double-differential cross section $\mathrm{d}^2\sigma /\mathrm{d}{}m\,\mathrm{d}|y |$ measurement

The pre-FSR cross section in bins of the dilepton invariant mass and the absolute value of the dilepton rapidity is measured according to

5 $$\begin{aligned} \sigma ^{ij}_\mathrm {det} = \frac{N_\mathrm {u}^{ij}}{\epsilon ^{ij}L_\text {int}}. \end{aligned}$$

The quantities $N_\mathrm {u}^{ij}$ and $\epsilon ^{ij}$ are defined in a given bin $(i,j)$, with $i$ corresponding to the binning in dilepton invariant mass and $j$ corresponding to the binning in absolute rapidity. The results are divided by the dilepton absolute rapidity bin widths $\Delta y^{j}$. The acceptance correction to the full phase space is not applied to the measurement, in order to keep theoretical uncertainties to a minimum.

**Fig. 4:**
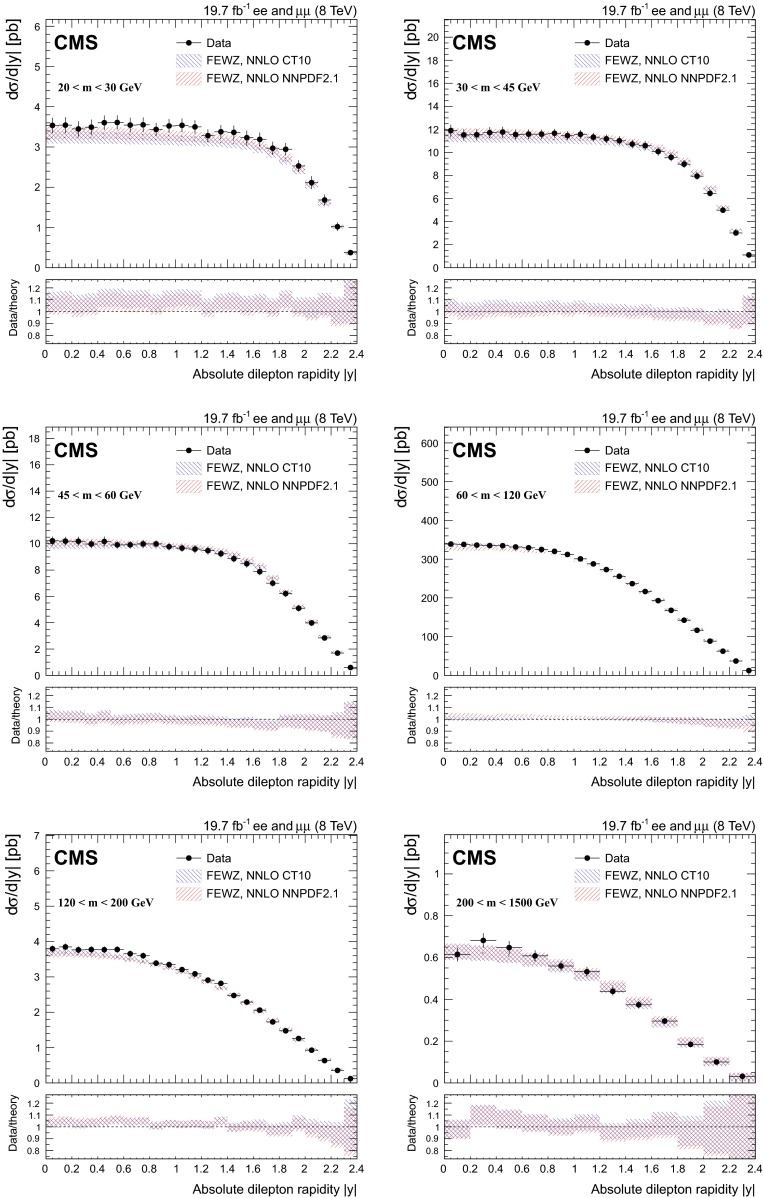
The DY dilepton rapidity distribution $\mathrm{d}\sigma /\mathrm{d}|y |$ within the detector acceptance, plotted for different mass ranges, as measured in the combined dilepton channel and as predicted by NNLO fewz  3.1 with CT10 PDF and NNLO NNPDF2.1 PDF calculations. There are six mass bins between 20 and 1500$\,\text {GeV}$, from *left* to *right* and from *top* to *bottom*. The uncertainty bands in the theoretical predictions combine the statistical and PDF uncertainties (*shaded bands*); the latter contributions are dominant

The $\chi ^2$ probability characterizing the consistency of the double-differential cross section measurements in the two channels is 45 % in the entire invariant mass-rapidity range of study. The measurements in the two channels are thus in agreement and are combined using the same procedure as for the differential cross sections described earlier in the section. Figure 4 shows the rapidity distribution $\mathrm{d}\sigma /\mathrm{d}|y |$ measured in the combined dilepton channel with the prediction by fewz  3.1 with the CT10 and NNPDF2.1 NNLO PDF sets. The cross section is evaluated within the detector acceptance and is plotted for six different mass ranges.

The uncertainty bands in the theoretical expectations include the statistical and the PDF uncertainties from the fewz  3.1 calculations summed in quadrature. The statistical uncertainty is significantly smaller than the PDF uncertainty, which is the dominant uncertainty in the fewz  3.1 calculations. In general, the PDF uncertainty assignment is different for each PDF set. The CT10 PDF uncertainties correspond to 90 % CL; to permit a consistent comparison with NNPDF2.1 the uncertainties are scaled to 68 % CL.

In the low-mass region, the results of the measurement are in better agreement with the NNPDF2.1 NNLO than with the CT10 NNLO estimate, which is systematically lower than NNPDF2.1 NNLO in that region. The $\chi ^2$ probability calculated between data and the theoretical expectation with total uncertainties on the combined results in the low-mass region is 16 % (76 %) for the CT10 (NNPDF2.1) PDFs. In the $\mathrm{Z}$ peak region, the two predictions are relatively close to each other and agree well with the measurements. The statistical uncertainties in the measurements in the highest mass region are of the order of the PDF uncertainty. The corresponding $\chi ^2$ probability calculated in the high mass region is 37 % (35 %) for the CT10 (NNPDF2.1) PDFs.

### Double ratio measurements

The ratios of the normalized differential and double-differential cross sections for the DY process at the center-of-mass energies of 7 and 8$\,\text {TeV}$ in bins of dilepton invariant mass and dilepton absolute rapidity are presented. The pre-FSR double ratio in bins of invariant mass is calculated following the prescription introduced in  [11] according to

6 $$\begin{aligned} R(\mathrm {p}\mathrm {p} \rightarrow \gamma ^{*}/\mathrm{Z}\rightarrow \ell ^{+}\ell ^{-}) = \frac{\left( \frac{1}{\sigma _{\mathrm{Z}}}\frac{\mathrm{d}\sigma }{\mathrm{d}{}m}\right) (8\,\text {TeV})}{\left( \frac{1}{\sigma _{\mathrm{Z}}}\frac{\mathrm{d}\sigma }{\mathrm{d}{}m}\right) (7\,\text {TeV})}, \end{aligned}$$

while the pre-FSR double ratio in bins of mass and rapidity is calculated as

7 $$\begin{aligned}&R_{\mathrm {det}}(\mathrm {p}\mathrm {p} \rightarrow \gamma ^{*}/\mathrm{Z}\rightarrow \ell ^{+}\ell ^{-}) \nonumber \\&\quad =\frac{\left( \frac{1}{\sigma _{\mathrm{Z}}}\frac{\mathrm{d}^2\sigma }{\mathrm{d}{}m\,\mathrm{d}|y |}\right) (8\,\text {TeV}, p_{\mathrm {T}} > 10,\,20\,\text {GeV})}{\left( \frac{1}{\sigma _{\mathrm{Z}}}\frac{\mathrm{d}^2\sigma }{\mathrm{d}{}m\,\mathrm{d}|y |}\right) (7\,\text {TeV}, p_{\mathrm {T}} > 9,\,14\,\text {GeV})}, \end{aligned}$$

where $\sigma _{\mathrm{Z}}$ is the cross section in the $\mathrm{Z}$ peak region; $\ell$ denotes $\mathrm {e}$ or $\mu$. The same binning is used for differential measurements at 7 and 8$\,\text {TeV}$ in order to compute the ratios consistently.

The double ratio measurements provide a high sensitivity to NNLO QCD effects and could potentially yield precise constraints on the PDFs; the theoretical systematic uncertainties in the cross section calculations at different center-of-mass energies have substantial correlations, as discussed in Sect. 9. Due to cancellation in the double ratio, the effect of the $\gamma \gamma$-initiated processes is negligible.

**Fig. 5:**
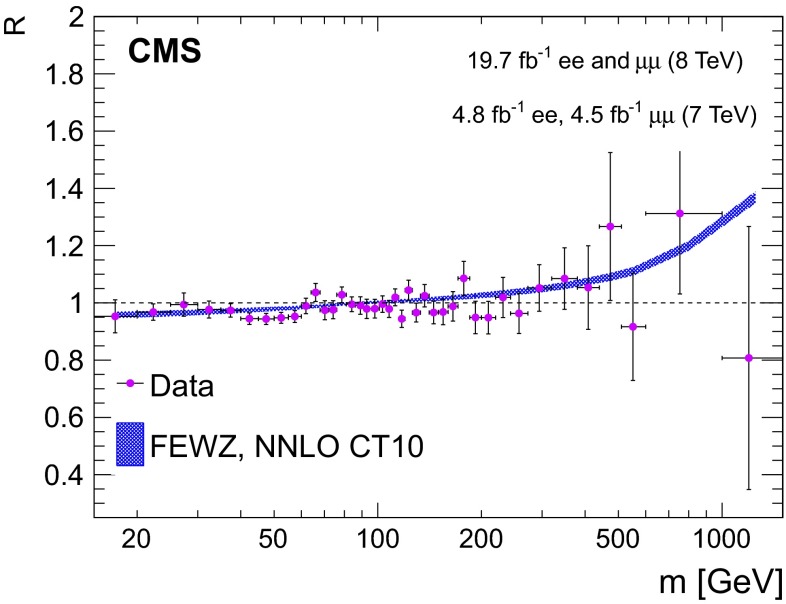
Measured DY double ratios at center-of-mass energies of 7 and 8$\,\text {TeV}$ in the combined dilepton channel as compared to NNLO fewz  3.1 calculations obtained with CT10 NNLO PDF, for the full phase space. The uncertainty band in the theoretical predictions combine the statistical and PDF uncertainties; the latter contributions are dominant. The exact definition of $R$ is given in Eq. (6)

Figure 5 shows the pre-FSR DY double ratio measurement in the combined (dielectron and dimuon) channel as a function of dilepton invariant mass, for the full phase space.

The theoretical prediction for the double ratio is calculated using fewz  3.1 with the CT10 NNLO PDF set. The shape of the distribution is defined entirely by the $\sqrt{s}$ and the Bjorken $x$ dependencies of the PDFs, since the dependence on the hard scattering cross section is canceled out. In the $\mathrm{Z}$ peak region, the expected double ratio is close to 1 by definition. It increases linearly as a function of the logarithm of the invariant mass in the region below 200$\,\text {GeV}$, where partons with small Bjorken $x$ contribute the most. The difference in regions of $x$ probed at 7 and 8$\,\text {TeV}$ center-of-mass energies leads to a rapid increase of the double ratio as a function of mass above 200$\,\text {GeV}$.

The uncertainty bands in the theoretical prediction of the double ratio include the statistical and the PDF uncertainties from the fewz  3.1 calculations summed in quadrature. The experimental systematic uncertainty calculation is described in Sect. 9.

We observe agreement of the double ratio measurement with the CT10 NNLO PDF theoretical prediction within uncertainties. The $\chi ^2$ probability from a comparison of the predicted and measured double ratios is 87 % with 40 degrees of freedom, calculated with the total uncertainties. At high mass, the statistical component of the uncertainty becomes significant, primarily from the 7$\,\text {TeV}$ measurements.

**Fig. 6:**
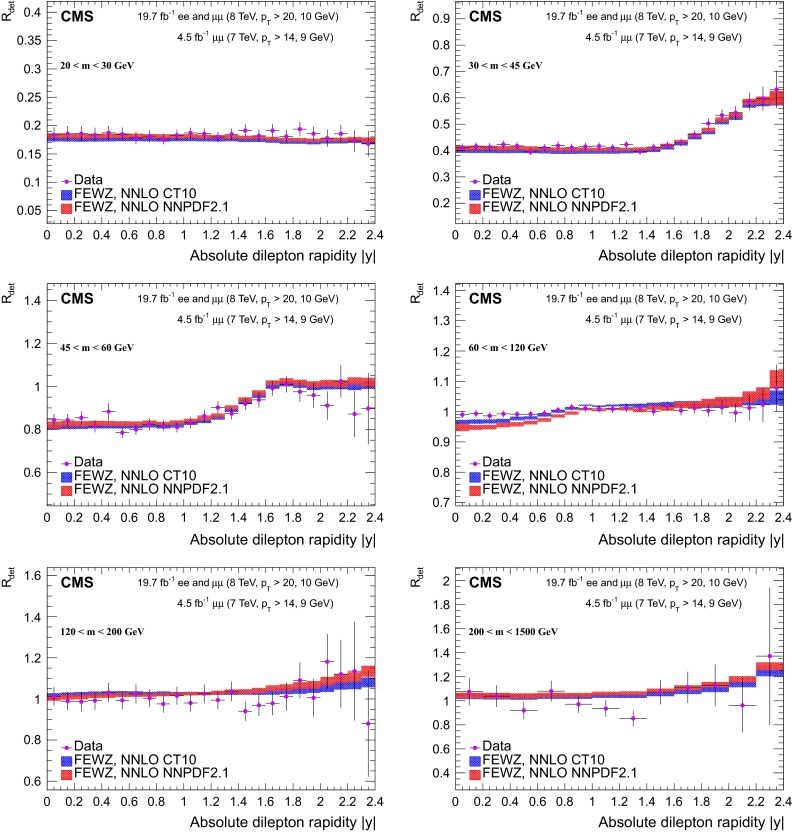
Measured DY double ratios as a function of the absolute dilepton rapidity within the detector acceptance, at center-of-mass energies of 7 and 8$\,\text {TeV}$, plotted for different mass ranges and as predicted by NNLO fewz  3.1 with CT10 and NNPDF2.1 NNLO PDF calculations. There are six mass bins between 20 and 1500$\,\text {GeV}$, from *left* to *right* and from *top* to *bottom*. The uncertainty bands in the theoretical predictions combine the statistical and PDF uncertainties (*shaded bands*); the latter contributions are dominant. The exact definition of $R_{\mathrm {det}}$ is given in Eq. (7)

The double ratios within the CMS acceptance as measured and as predicted by fewz  3.1 CT10 and NNPDF2.1 NNLO PDF calculations as a function of dilepton rapidity in six mass bins are summarized in Fig. 6. The measurements having the smallest experimental systematic uncertainty are used in the calculation. Thus, the 8$\,\text {TeV}$ measurement entering the numerator is estimated in the combined channel, while the 7$\,\text {TeV}$ measurement in the denominator is estimated in the dimuon channel [12].

The shape of the theoretical prediction of the double ratio is nearly independent of the dilepton rapidity at low mass, showing an increase as a function of rapidity by up to 20 % in the $\mathrm{Z}$ peak region and at high mass, and a significant dependence on rapidity in the 30–60$\,\text {GeV}$ region. The uncertainty bands in the theoretical predictions of the double ratio include the statistical and the PDF uncertainties from the fewz 3.1 calculations summed in quadrature. The uncertainties related to QCD evolution scale dependence are evaluated by varying the renormalization and factorization scales simultaneously between the values 2$m$, $m$, and $m$/2, with $m$ corresponding to the middle of the invariant mass bin. The scale variation uncertainties reach up to 2 % and are included in the theoretical error band.

The double ratio predictions calculated with the CT10 NNLO and NNPDF2.1 NNLO PDFs agree with the measurements. Below the $\mathrm{Z}$ peak, NNPDF2.1 NNLO PDF theoretical predictions are in a closer agreement with the measurement. In the $\mathrm{Z}$ peak region, a difference in the slope of both theoretical predictions as compared to the measurement is observed in the central absolute rapidity region. In the high-rapidity and high-mass regions, the effect of the limited number of events in the 7$\,\text {TeV}$ measurement is significant. In the 120–200$\,\text {GeV}$ region, the measurement is at the lower edge of the uncertainty band of the theory predictions.

The DY double-differential cross section and double ratio measurements presented here can be used to impose constraints on the quark and antiquark PDFs in a wide range of $x$, complementing the data from the fixed-target experiments with modern collider data.

## Summary

This paper presents measurements of the Drell–Yan differential cross section $\mathrm{d}\sigma /\mathrm{d}{}m$ and the double-differential cross section $\mathrm{d}^2\sigma /\mathrm{d}{}m\,\mathrm{d}|y |$ with proton–proton collision data collected with the CMS detector at the LHC at a center-of-mass energy of $8\,\text {TeV}$. In addition, the first measurements of the ratios of the normalized differential and double-differential cross sections for the DY process at center-of-mass energies of 7 and 8$\,\text {TeV}$ in bins of dilepton invariant mass and absolute rapidity are presented. A previously published CMS measurement based on 7$\,\text {TeV}$ data [12] is used for the double ratio calculations.

The measured inclusive cross section in the $\mathrm{Z}$ peak region is $1138 \pm 8\,\text {(exp)} \pm 25\,\text {(theo)} \pm 30\,\text {(lumi)} \text {\,pb}$ for the combination of the dielectron and dimuon channels. This is the most precise measurement of the cross section in the $\mathrm{Z}$ peak region at $\sqrt{s} = 8\,\text {TeV}$ in CMS. The $\mathrm{d}\sigma /\mathrm{d}{}m$ and $\mathrm{d}^2\sigma /\mathrm{d}{}m\,\mathrm{d}|y |$ measurements agree with the NNLO theoretical predictions computed with fewz  3.1 using the CT10 NNLO and NNPDF2.1 NNLO PDFs. The double ratio measurement agrees with the theoretical prediction within the systematic and PDF uncertainties.

The experimental uncertainties in the double-differential cross section and the double ratio measurements presented are relatively small compared to the PDF uncertainties.
